# A dominant negative *Kcnd3* F227del mutation in mice causes spinocerebellar ataxia type 22 (SCA22) by impairing ER and Golgi functioning

**DOI:** 10.1002/path.6368

**Published:** 2024-11-19

**Authors:** Hao‐Chih Hung, Jia‐Han Lin, Yuan‐Chi Teng, Cheng‐Heng Kao, Pei‐Yu Wang, Bing‐Wen Soong, Ting‐Fen Tsai

**Affiliations:** ^1^ Department of Life Sciences and Institute of Genome Sciences National Yang Ming Chiao Tung University Taipei Taiwan; ^2^ Department of Medical Research Taipei Veterans General Hospital Taipei Taiwan; ^3^ Center of General Education Chang Gung University Taoyuan Taiwan; ^4^ Graduate Institute of Brain and Mind Sciences, College of Medicine National Taiwan University Taipei Taiwan; ^5^ Department of Neurology Taipei Veterans General Hospital Taipei Taiwan; ^6^ Brain Research Center National Yang Ming Chiao Tung University Taipei Taiwan; ^7^ Center for Healthy Longevity and Aging Sciences National Yang Ming Chiao Tung University Taipei Taiwan; ^8^ National Health Research Institutes Institute of Molecular and Genomic Medicine Miaoli Taiwan

**Keywords:** spinocerebellar ataxia type 22, KCND3, dominant negative mutation, Purkinje cells, endoplasmic reticulum and Golgi, trafficking defect

## Abstract

Spinocerebellar ataxia type 22 (SCA22) caused by *KCND3* mutations is an autosomal dominant disorder. We established a mouse model carrying the *Kcnd3* F227del mutation to study the molecular pathogenesis. Four findings were pinpointed. First, the heterozygous mice exhibited an early onset of defects in motor coordination and balance which mirror those of SCA22 patients. The degeneration and a minor loss of Purkinje cells, together with the concurrent presence of neuroinflammation, as well as the previous finding on electrophysiological changes, may all contribute to the development of the SCA22 ataxia phenotype in mice carrying the *Kcnd3* F227del mutant protein. Second, the mutant protein is retained by the endoplasmic reticulum and Golgi, leading to activation of the unfolded protein response and a severe trafficking defect that affects its membrane destination. Intriguingly, profound damage of the Golgi is the earliest manifestation. Third, analysis of the transcriptome revealed that the *Kcnd3* F227del mutation down‐regulates a panel of genes involved in the functioning of synapses and neurogenesis which are tightly linked to the functioning of Purkinje cells. Finally, no ataxia phenotypes were detectable in knockout mice carrying a loss‐of‐function *Kcnd3* mutation. Thus, *Kcnd3* F227del is a dominant‐negative mutation. This mouse model may serve as a preclinical model for exploring therapeutic strategies to treat patients. © 2024 The Author(s). *The Journal of Pathology* published by John Wiley & Sons Ltd on behalf of The Pathological Society of Great Britain and Ireland.

## Introduction

Spinocerebellar ataxia (SCA) encompasses a highly heterogeneous group of dominantly inherited neurological disorders that are characterized by their clinical, pathological, and genetic diversity. The hallmark of SCA is the progressive degeneration of cerebellar function, often accompanied by varying degrees of impairment across other neurological systems [1]. The world‐wide prevalence of SCA is approximately three cases per million individuals. Over 40 genetic types of SCA have been identified, each associated with distinct genetic mutations, such as glutamine‐encoding CAG repeat expansions (polyQ), DNA rearrangements, missense mutations, and insertions or deletions [2]. While the causative genes of SCA exhibit genetic heterogeneity, the affected cellular processes are largely similar and primarily involve dysfunction of Purkinje cells, which leads to reduced intrinsic firing and subsequently motor defects [2, 3].

Purkinje cells are distinct from other types of neurons. Anatomically, Purkinje cells serve as single output neurons. They receive and integrate excitatory signals from parallel fibers and climbing fibers, as well as inhibitory signals from interneurons. These cells then transmit inhibitory signals to GABAergic inputs in the deep cerebellar nuclei [2, 3]. Morphologically, the dendrites of Purkinje cells are remarkably extensive and branched. Physiologically, Purkinje cells function as fast pacemakers and fire spontaneous action potentials at a high frequency in a manner that is coordinated by numerous ion channels. Sodium channels within the axon initial segment govern the simple spike of an action potential and are critical for sustained firing, while dendrite calcium channels control complex firing and sustained pacemaking. Dendrite and axon potassium channels participate in spike frequency filtering [4, 5, 6]. Dysfunction of these ion channels often leads to the development of neurodegenerative diseases, a condition referred to as channelopathy.

*KCND3* (potassium voltage‐gated channel subfamily D member 3) is the disease gene of SCA19 and SCA22. In 2012, *KCND3* mutations, including T352P, M373I, and S390N, were discovered and assigned to SCA19 [7]. Meanwhile, the *KCND3* mutations of F227del, V338E, G345V, and T377M were assigned to SCA22 [8]. Since then, more mutations within the *KCND3* gene have been found to be associated with SCA, including A293Fdup, C317Y, P375S, G384, L45P, and P614S. Notably these mutations are located within the 5' region of the *KCND3* gene and seem to reduce channel activity and ion conductance [7, 8, 9, 10, 11]. The *KCND3* gene encodes Kv4.3, which is an alpha subunit of the Shal family of A‐type voltage‐gated potassium channels and plays a vital role in the repolarization phase of action potentials in excitable cells [8]. In mammals, Kv4.3 is highly expressed in the brain and cerebellum, in particular in Purkinje cells, granule cells, Lugaro cells, stellate cells, and basket cells [12, 13]. Similar to other voltage‐gated potassium channels, Kv4.3 assembles as a homotetramer or, alternatively, as a heterotetramer with other channels belonging to the Shal subfamily. Each alpha subunit is composed of six transmembrane segments (S1–S6) along with a re‐entrant loop that connects S5 and S6. The voltage‐sensing domain is formed by S1–S4, while the ion‐selective pore is formed by S5, S6, and the re‐entrant loop [14]. The *KCND3* F227del mutation was originally found in Taiwanese and French families [8]. The amino acid at the 227th residue, namely phenylalanine (F), is located within the S2 transmembrane domain of Kv4.3. This domain is conserved between Kv4 and Kv1 channels, implying that integrity of this region plays a crucial role in maintaining the normal functions of the channel. Previous *in vitro* experiments have indicated that the *Kcnd3* F227del mutation reduces the potassium current, possibly due to endoplasmic reticulum (ER) retention of the Kv4.3 mutant protein [8, 15]. However, the *in vivo* molecular mechanism has remained elusive. Here, we establish a knock‐in (KI) mouse model that carries the *Kcnd3* F277del mutation to elucidate the pathogenesis and molecular mechanism of the phenotype caused by this mutation.

## Materials and methods

### The *Kncd3* F227del knock‐in (KI) and knockout (KO) mice

The KI allele of *Kcnd3* F227del was generated by homologous recombination, originally in the B6N ES cell background, and later the line was backcrossed with C57BL/6J mice (supplementary material, Figure S1A–D). Only congenic mice (*N* > 10) were used. The *Kcnd3* KO null allele was generated using the CRISPR/Cas9 system in the C57BL/6J background; the founder mice were bred with C57BL/6J mice for four generations to eliminate any potential off‐target effects. All mice were housed in a specific pathogen‐free facility with a 12/12 h light/dark cycle at a controlled temperature (20–22 °C) at the Laboratory Animal Center, National Yang Ming Chiao Tung University. All of the animal protocols used adhered to the recommendations outlined in the ‘Guide for the Care and Use of Laboratory Animals’ (National Academy Press, Washington, DC, USA). The Institutional Animal Care and Use Committees of the National Yang Ming Chiao Tung University approved this study (Nos 1060709 and 1080614).

Detailed methods are provided in Supplementary materials and methods.

## Results

### The *Kcnd3* F227del mice display defects in motor coordination and balance

**Figure 1:**
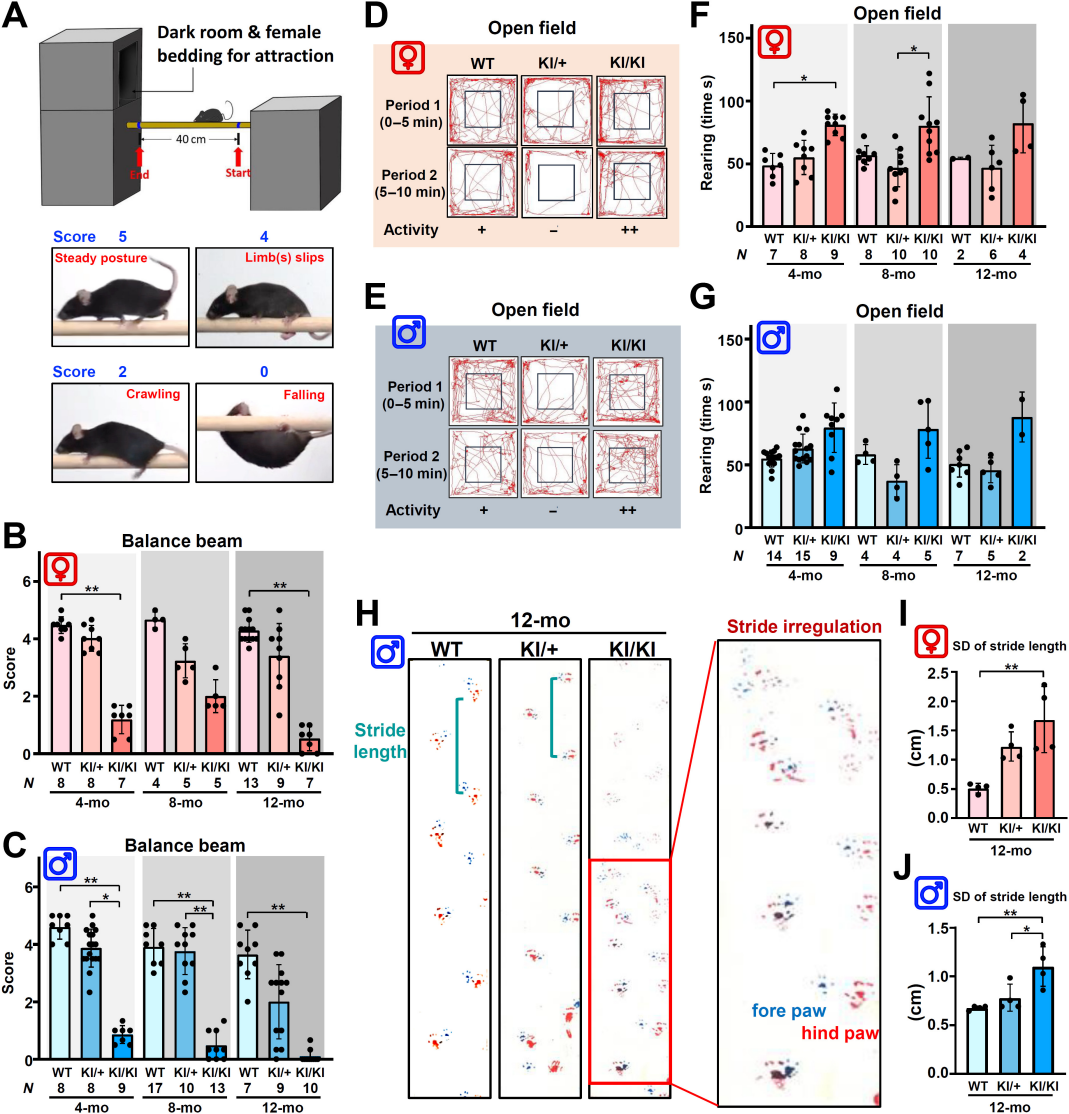
The *Kcnd3* F227del mutation affects motor coordination and causes balance defects that resemble the phenotype of human SCA22 patients. (A) Device setup and the scoring system for the balance beam assay. (B and C) The performance scores of the balance bean assay obtained from both female and male WT, *Kcnd3* F227del KI/+, and KI/KI mice at 4, 8, and 12 months old are shown. (D and E) Representative walking tracks of female and male mice at 8 months old over the first 5 min (Period 1) and next 5 min (Period 2) obtained using the open field assay. The activity levels of WT, *Kcnd3* F227del KI/+, and KI/KI mice are denoted as active (++), moderately active (+), or less active (−). (F and G) The frequency of rearing in female and male WT, *Kcnd3* F227del KI/+, and KI/KI mice during the open field assay. (H) Representative images from the footprint assay, highlighting the paw discordance of male *Kcnd3* KI/KI mice at 12 months old. (I, J) The variation (SD) of stride length of the same paws for female (I) and male (J) mice. The stride length was measured as the distance between the tops of the same palm between two steps using ImageJ (NIH, Bethesda, MD, USA). The results were obtained from WT, *Kcnd3* F227del KI/+, and KI/KI mice at 12 months of age. For each mouse, two fore paws and two hind paws were analyzed. Mean ± SD. Statistical analyses were performed using the Kruskal–Wallis test (B, C, F, and G) and one‐way ANOVA corrected with Bonferroni's multiple comparison test (I and J) in order to assess statistical differences. *p < 0.05; **p < 0.01.

We generated a KI mouse model carrying the *Kcnd3* F227del allele in order to investigate the phenotypic effects of this mutation (supplementary material, Figure S1A–D). We conducted behavioral tests to assess the motor coordination and motor activity of heterozygous (KI/+) and homozygous (KI/KI) *Kcnd3* F227del mice at different ages, namely 4, 8, and 12 months. The balance beam walking test, which is a sensitive and well‐established evaluation of motor coordination and balance [16], was used to examine the phenotype (Figure 1A). Female and male KI/+ mice displayed signs of impaired balance, including limb slippage and crawling behavior, at 8 and 12 months of age, respectively (Figure 1B,C). Notably, both sexes of KI/KI mice displayed more severe coordination abnormalities at 4 months of age (Figure 1B,C and supplementary material, Video S1).

In addition, the open field assay provided evidence of early‐onset abnormalities in motor function (supplementary material, Figure S1E). Interestingly, we observed a reduced exploratory activity in KI/+ mice, while KI/KI mice exhibited a higher exploratory activity (Figure 1D,E). Additionally, female KI/KI mice displayed increased rearing time at 4 months of age (Figure 1F,G). Furthermore, female KI/KI mice showed an increased travel distance at 8 and 12 months of age, while male KI/KI mice only exhibited a significant difference in this parameter at 4 months of age (supplementary material, Figure S1F,G). Notably, both KI/+ and KI/KI mice displayed abnormal jumping behavior at a much higher frequency than did WT mice (supplementary material, Video S2), which may indicate the presence of underlying brain function abnormalities. Interestingly, hyperactivity and alterations in walking trajectory were observed in the KI/KI mice (Figure 1D–G), which is similar to the findings observed in Kv3.3 KO mice and Kv3.1 and Kv3.3 double KO mice [17]. However, the underlying mechanism behind this phenotype remains unclear. Next, we performed gait analysis by collecting each mouse's ambulating footprints. Typical results for the gait tests of ataxia mice include decreased stride length and increased stance width [18]. At 12 months old, the KI/+ mice were found to have a slightly shorter stride length and a herringbone walking path, both of which resemble a mild ataxia phenotype (Figure 1H). The KI/KI mice had a more severe ataxia phenotype, which included an irregular and unequal gait, as revealed by a significant difference in the variation of stride length between steps (Figure 1I,J), as well as dissonance between the fore‐paw and hind‐paw prints (Figure 1H). The ambulating footprints of KI/+ and KI/KI mice mirrored the typical pattern for an ataxia phenotype. Importantly, the ataxia phenotype observed in the KI/+ and KI/KI mice was not associated with body weight loss (supplementary material, Figure S1H,I). Taken together, the behavior analyses revealed that the *Kcnd3* F227del mutation indeed causes prominent defects in motor coordination and balance and that this KI mouse model exhibits a dosage‐dependent ataxia phenotype; namely, a more severe defect was detected in the KI/KI mice compared with the KI/+ mice.

### The *Kcnd3* F227del mutation results in degeneration and a minor loss of Purkinje cells as well as neuroinflammation in the cerebellum

**Figure 2:**
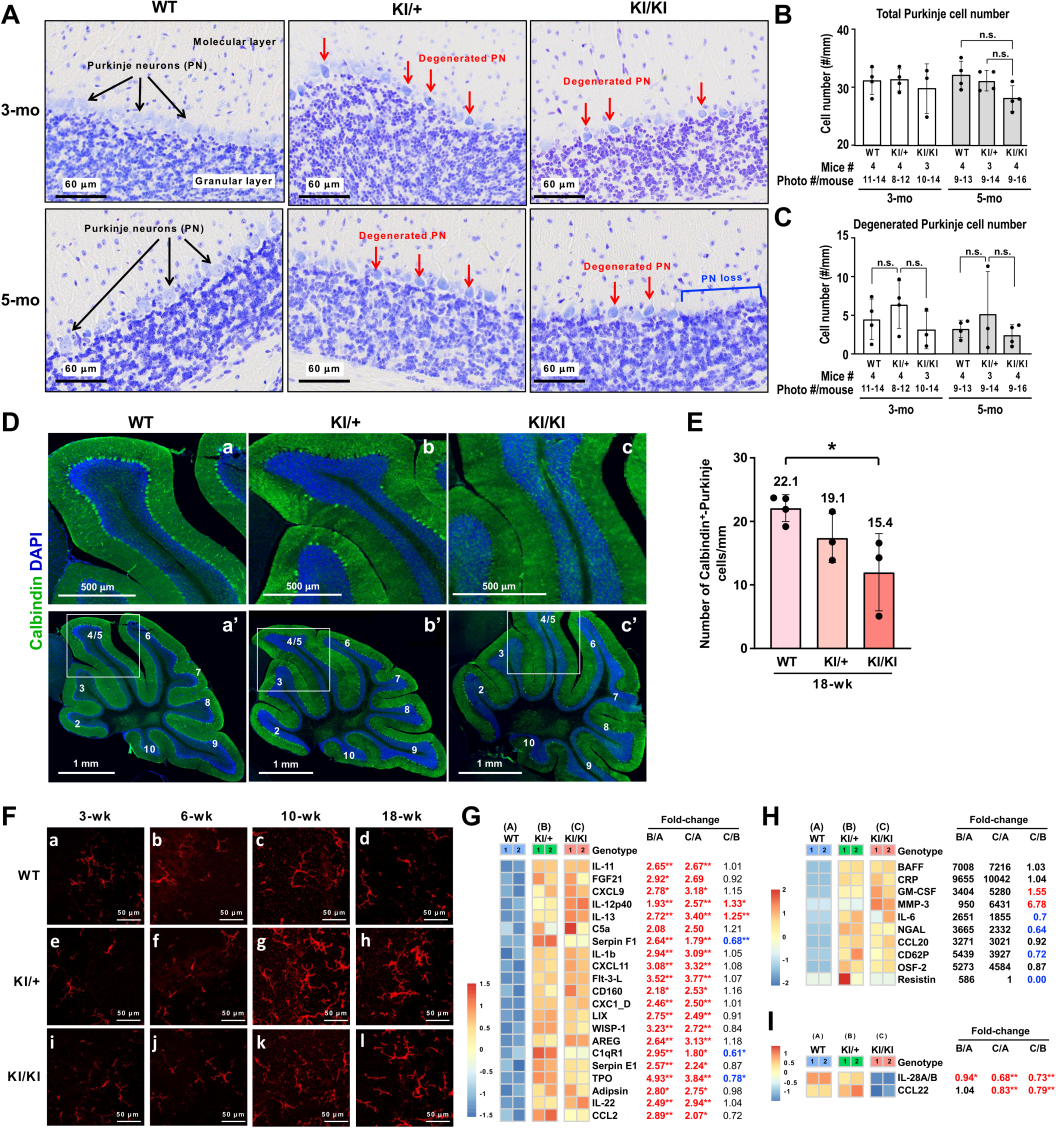
Degeneration and a minor loss of Purkinje cells and the presence of neuroinflammation in the cerebellums of *Kcnd3* F227del KI mice. (A) Representative images of cresyl violet‐stained cerebellums of WT, *Kcnd3* F227del KI/+, and KI/KI mice at 3 and 5 months of age. Black arrow, Purkinje neuron; red arrow, degenerated Purkinje neuron (PN). (B) Quantitation of Purkinje cell numbers per mm from the cerebellums of the WT, *Kcnd3* F227del KI/+, and KI/KI mice at 3 and 5 months of age. Each dot represents the average of the Purkinje cell number, which was normalized against the length of the junction between the molecular and granular layers; these averages were obtained from assessment of cresyl violet‐stained cell photographs from a single individual mouse. (C) Quantitation of degenerated Purkinje cell numbers per mm from the cerebellums of the WT, *Kcnd3* F227del KI/+, and KI/KI mice at 3 and 5 months of age. Each dot represents the average of the degenerated Purkinje cell number, which was normalized against the length of the junction between the molecular and granular layers; these averages were obtained from cresyl violet‐stained cell photographs from a single individual mouse. (D) Immunofluorescence staining for calbindin, a Purkinje cell marker. (a–c) Representative images of the cerebellums of WT, *Kcnd3* F227del KI/+, and KI/KI female mice at 18 weeks old. (a′–c′) The gross view of the images is shown in a–c. The boxed areas indicate the magnified regions. Numbers correspond to the cerebellar lobe. (E) Quantification of calbindin‐positive Purkinje cells per mm in the cerebellums of WT, *Kcnd3* F227del KI/+, and KI/KI female mice at 18 weeks of age. (F) Immunofluorescence staining for IbaI to detect microglia in the cerebellar cortices of WT, *Kcnd3* F227del KI/+, and KI/KI mice at 3, 6, 10, and 18 weeks of age. (G–I) Heatmaps of the signal intensities obtained from cytokine array analysis using cerebellum lysates of WT, *Kcnd3* F227del KI/+, and KI/KI mice at 10 weeks old. Red and blue indicate a significant increase and a significant decrease in signal, respectively. There are two samples for each genotype. In each sample, equal amounts of the cerebellum homogenate from a male and a female, namely 100 μg of homogenate from a male sample and 100 μg of homogenate from a female sample, were pooled to avoid a potential sex‐specific effect on cytokine profiling. Mean ± SD. In B, C, E, G, H, and I, statistical analyses were performed using one‐way ANOVA corrected with Bonferroni's multiple comparison test. *p < 0.05; **p < 0.01.

To better understand the pathophysiology caused by the *Kcnd3* F227del mutation, we focused on the cerebellum, where *Kcnd3* is highly expressed in Purkinje cells. Firstly, we utilized cresyl violet staining, which is often used to detect neurodegeneration [19], to examine the cerebellar pathology of *Kcnd3* F227del mice. A trend of a decrease in the number of Purkinje cells in the KI/KI mice was observed at 5 months of age (Figure 2A,B). In the KI/+ mice, we observed an increased presence of Purkinje cells that looked shrunken and appeared darker in the cresyl violet staining, i.e. showed some signs of degeneration (red arrows in Figure 2A). We will call these cells ‘degenerated Purkinje cells’; we found a trend of an increase of these cells in the KI/+ mice at both 3 and 5 months of age (Figure 2C). Additionally, using immunofluorescence staining of calbindin, a Purkinje cell marker, it was possible to detect a significant loss of calbindin‐positive Purkinje cells in the KI/KI mice at 18 weeks of age (an age close to 5 months) (Figure 2D,E); this mirrors a characteristic feature of SCA, namely the loss of calbindin‐positive Purkinje cells, in both human patients [20] and in other mouse models [2]. Notably, neuroinflammation, as indicated by an increase in Iba1‐positive microglia with a large cell body and extended processes [21, 22, 23], was evident in the cerebellar cortices of KI/+ and KI/KI mice after 10 weeks of age (Figure 2F). To validate the immune response, we performed a cytokine array analysis using cerebellum lysates of 10‐week‐old mice. An increase in 31 different cytokines was detected (Figure 2G–I and supplementary material, Figure S2), with nine of them showing substantial induction (Figure 2H). Among these cytokines, IL‐6 and IL‐1β are known to activate microglia‐mediated phagocytosis [24]. Interestingly, the cytokine levels of IL‐28A/B and CCL22 were decreased in the cerebellums of *Kcnd3* F227del KI/KI mice (Figure 2I). Together, these results reveal that the *Kcnd3* F227del mutation leads to degeneration and a limited loss of Purkinje cells and the concurrent presence of neuroinflammation.

### The *Kcnd3* F227del mutation causes degeneration of the Golgi apparatus, the mitochondria, and the ER

**Figure 3:**
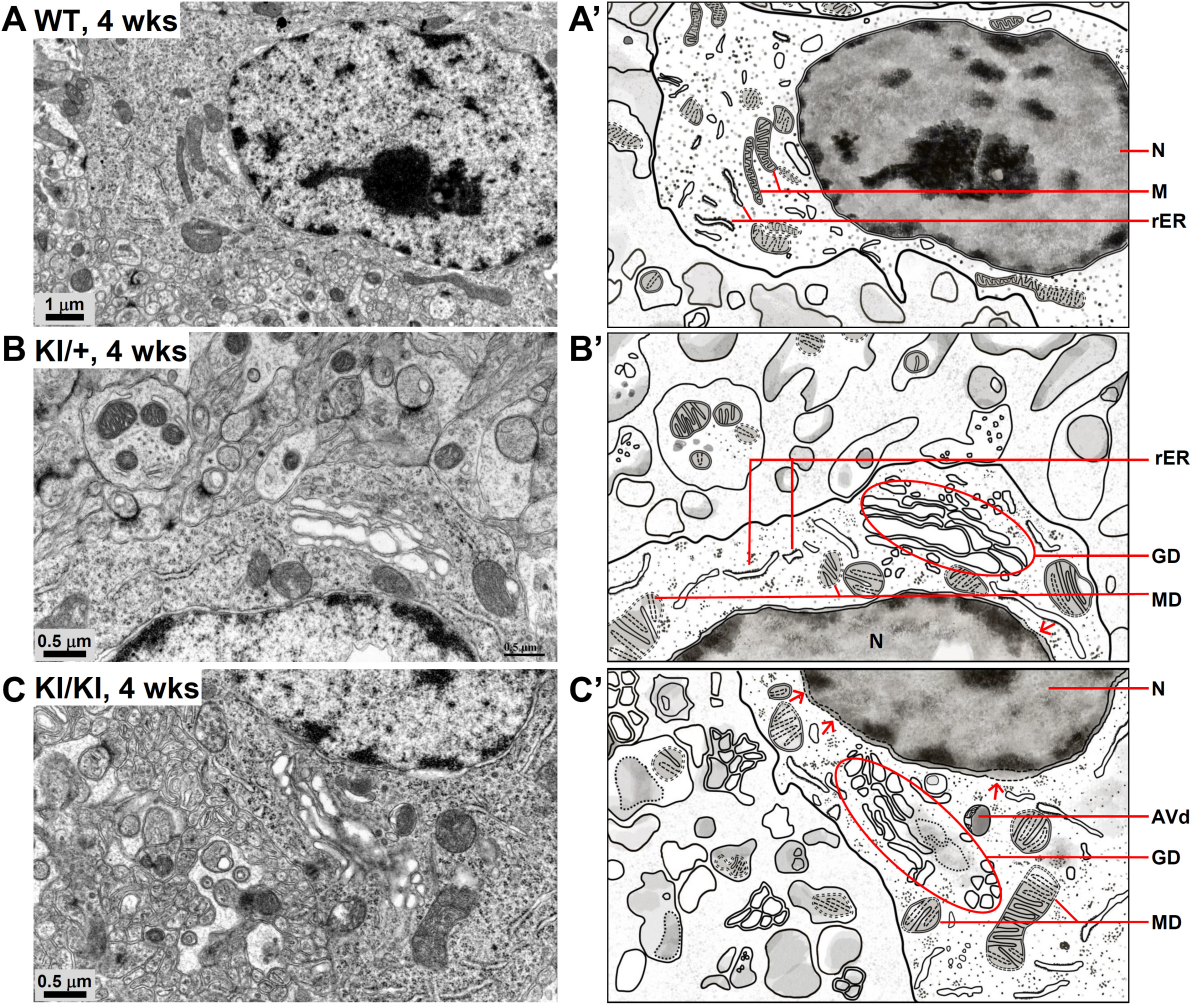
The *Kcnd3* F227del mutation causes obvious degeneration of the Golgi apparatus and mitochondria in the Purkinje cells at 4 weeks of age. (A–C) Representative TEM images of Purkinje cells in WT (A), *Kcnd3* F227del KI/+ (B), and KI/KI mice (C). (A′–C′) Schematic representation of the TEM images (1:1 ratio) shown in A–C. AVd, autolysosome; G, Golgi apparatus; GD, Golgi degeneration; M, mitochondria; MD, mitochondria degeneration; N, nucleus; rER, rough endoplasmic reticulum; red arrow, breakdown of nuclear envelope.

To examine the ultrastructural damage to Purkinje cells caused by the *Kcnd3* F227del mutation, we carried out TEM (transmission electron microscopy) analysis on mice at early ages, namely 4 and 6 weeks, before the development of any overt phenotype. In the Purkinje cells of WT mice at 4 weeks of age, the mitochondria had an elongated structure and the ER exhibited an organized structure (Figure 3A,A′). In the Purkinje cells of KI/+ mice at 4 weeks of age, however, degenerated mitochondria with a more rounded shape were observed. In addition, some of the mitochondria lacked an outer membrane. Strikingly, the Golgi apparatus exhibited signs of swelling, indicative of organelle degeneration (Figure 3B,B′). Remarkably, in the Purkinje cells of KI/KI mice at 4 weeks of age, there was an even more pronounced degeneration affecting both the Golgi and the mitochondria. This was characterized by a notable enlargement of the Golgi with a loose structure and by the mitochondria lacking inner membrane cristae (Figure 3C,C′). At 6 weeks of age, all of the ultrastructural damage in Purkinje cells was exacerbated in the KI/+ and KI/KI mice (supplementary material, Figure S3). Notably, there is a positive correlation between the dosage of the mutant Kcnd3 F227del protein and the degree of ultrastructural damage detected in Purkinje cells (supplementary material, Figure S4). In addition, obvious dilation of the ER, as well as degeneration and breakdown of the nuclear membrane, was detectable in the KI/KI mice (supplementary material, Figure S3). Importantly, among the organelle abnormalities, prominent Golgi damage appears to be the earliest manifestation in the Purkinje cells of both KI/+ and KI/KI mice (Figure 3B,C).

### The Kcnd3 F227del mutant protein is retained in the ER and Golgi apparatus, which leads to severe trafficking defects and the unfolded protein response (UPR)

**Figure 4:**
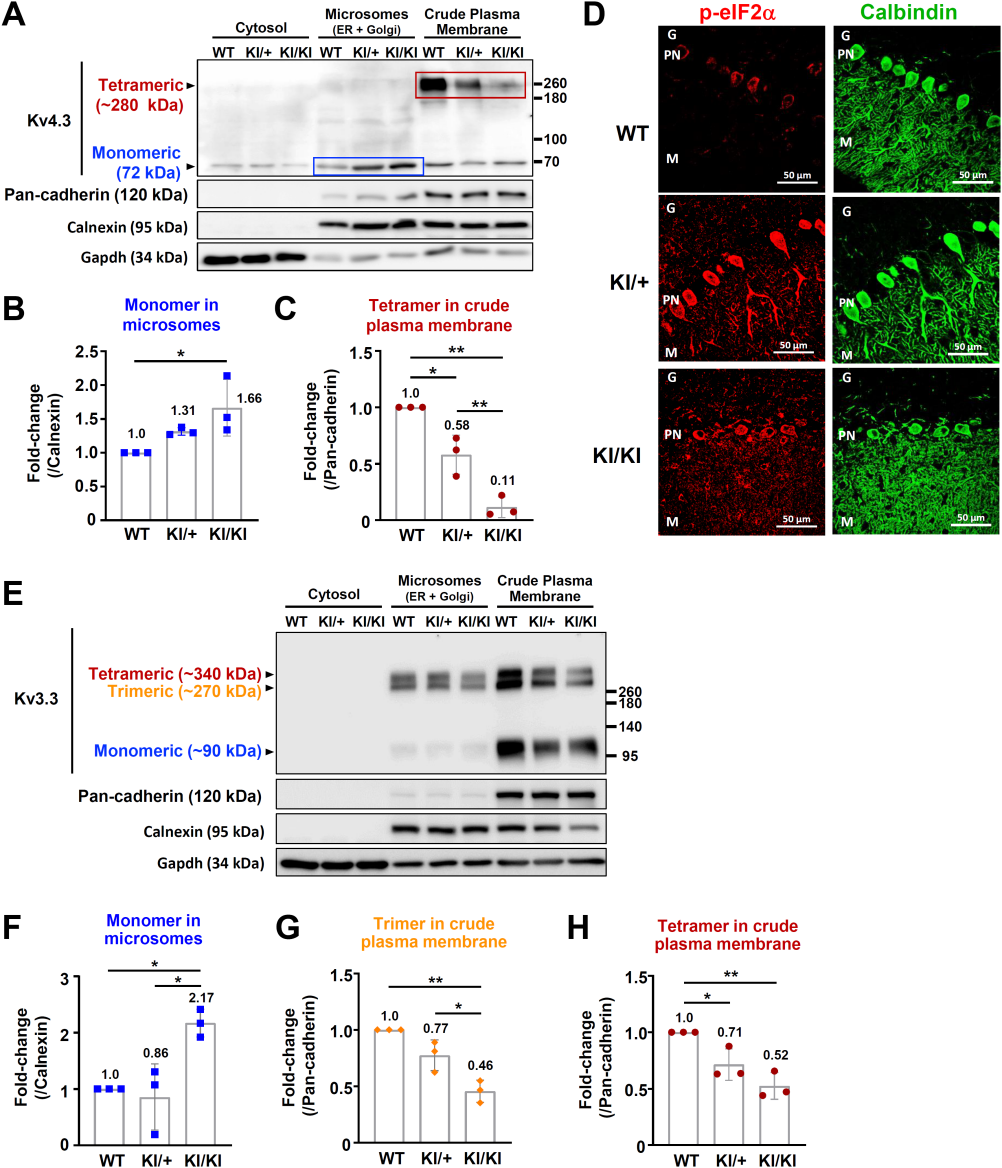
The monomer of mutant Kv4.3, namely the Kcnd3 F227del mutant protein, and Kv3.3 wild‐type protein are retained in the ER and Golgi fractions. (A) Western blot analyses of the Kcnd3 protein using extracts prepared by subcellular fractionation of the cerebellums of WT, *Kcnd3* F227del KI/+, and KI/KI mice at 6 weeks of age. Antibodies against pan‐cadherin (a marker for plasma membrane), calnexin (a marker for rER), and Gapdh (a marker for cytosol) were used for the western blotting analysis. (B) Quantification of Kcnd3 monomer levels in the microsome fractions. (C) Quantification of Kcnd3 tetramer levels in the plasma membrane fractions. (D) Immunofluorescence staining for phosphorylated eIF2a and calbindin in the cerebellums of WT, *Kcnd3* F227del KI/+, and KI/KI mice at 10 weeks of age. (E) Western blot analyses for the Kcnc3 protein using extracts prepared by subcellular fractionation of the cerebellums of WT, *Kcnd3* F227del KI/+, and KI/KI mice at 6 weeks of age. Antibodies against pan‐cadherin, calnexin, and Gapdh were used for the western blot analyses. (F) Quantification of Kcnc3 monomer levels in the microsome fractions. (G) Quantification of Kcnc3 trimer levels in the plasma membrane fractions. (H) Quantification of Kcnc3 tetramer levels in the plasma membrane fractions. Mean ± SD. In B and C, one‐way ANOVA corrected with Bonferroni's multiple comparison test was performed to analyze the statistical differences. *p < 0.05; **p < 0.01. G, granule layer; M, molecular layer; PN, Purkinje neuron.

Previous findings have suggested that the *Kcnd3* F227del mutation impairs the trafficking of the Kcnd3 protein toward the plasma membrane when there is *in vitro* overexpression of this mutant protein in non‐neuronal cells [8, 15]. To validate this finding *in vivo* in neurons, we conducted subcellular fractionation by dividing the mouse cerebellum lysate into three subcellular compartments, namely cytosol, microsomes (ER and Golgi), and crude plasma membrane (supplementary material, Figure S5A). We next determined the subcellular localization of Kv4.3 (Kcnd3 protein). In WT mice, Kv4.3 was predominantly found in the plasma membrane fraction as a tetramer. Strikingly, in *Kcnd3* F227del KI/+ and KI/KI mice, the mutant protein was obviously retained as a monomer in the microsome fraction; in addition, much less of the mutant Kv4.3 as a tetramer was detected in the plasma membrane fraction (Figure 4A). Quantification revealed that the level of F227del Kv4.3 monomer was significantly increased in the microsome fraction, while the level of tetramer mutant protein was significantly decreased in the plasma membrane fraction. Remarkably, in the KI/+ and KI/KI mice, about 58% and 11% of the Kv4.3 tetramer, respectively, were transported to the plasma membrane (Figure 4B,C). Since forming tetramers in the plasma membrane is essential to the functioning of Kv4.3, the impairment to the membrane destination of Kv4.3 caused by the F227del mutation will result in the channelopathy of Kv4.3.

Furthermore, the accumulation of mutant proteins in the ER can trigger activation of the UPR [25]. Indeed, activation of the UPR was detectable in the Purkinje cells of *Kcnd3* F227del KI/+ and KI/KI mice, as indicated by the prominent immunofluorescence staining of phosphorylated eIF2a (Figure 4D). However, it is worth noting that, at this stage, there are no changes detected in autophagy markers (supplementary material, Figure S5B–E). Additionally, in the *Kcnd3* F227del KI/+ and KI/KI mice, our immunofluorescence analysis revealed that in the neurons of the cerebellum, the F227del mutant Kv4.3 was also retained in the ER, as indicated by the co‐localization of the mutant protein with calnexin. Moreover, in neurons expressing the mutant protein, the immunofluorescence of the calnexin signal in the ER appeared to be greater in size, suggesting a phenotype associated with UPR‐induced ER swelling (supplementary material, Figure S6). These results suggest that in addition to Purkinje cells, other neurons expressing the F227del mutant Kv4.3 are likely to have similar defects in membrane trafficking.

Even more strikingly, we discovered that the membrane destination of Kv3.3, which is another potassium channel protein encoded by the *Kcnc3* gene, was impaired in the cerebellums of *Kcnd3* F227del KI/+ and KI/KI mice; the genotype of *Kcnc3* is wild type in these KI/+ and KI/KI mice. Genetic mutations of the *KCNC3* gene cause SCA13 [2]. Quantification analysis revealed that the levels of tetrameric and trimeric Kcnc3 proteins (Kv3.3) were significantly decreased in the plasma membrane fraction (Figure 4E–H), suggesting that the F227del mutant Kv4.3 has a destructive effect that damages the structure of the ER and Golgi, which then impairs organelle functioning. This, in turn, will indirectly lead to a trafficking defect that affects the membrane destination of other potassium channel proteins, such as Kv3.3.

### Transcriptomic analysis reveals that the Kcnd3 F227del mutant protein impairs neuronal functions

**Figure 5:**
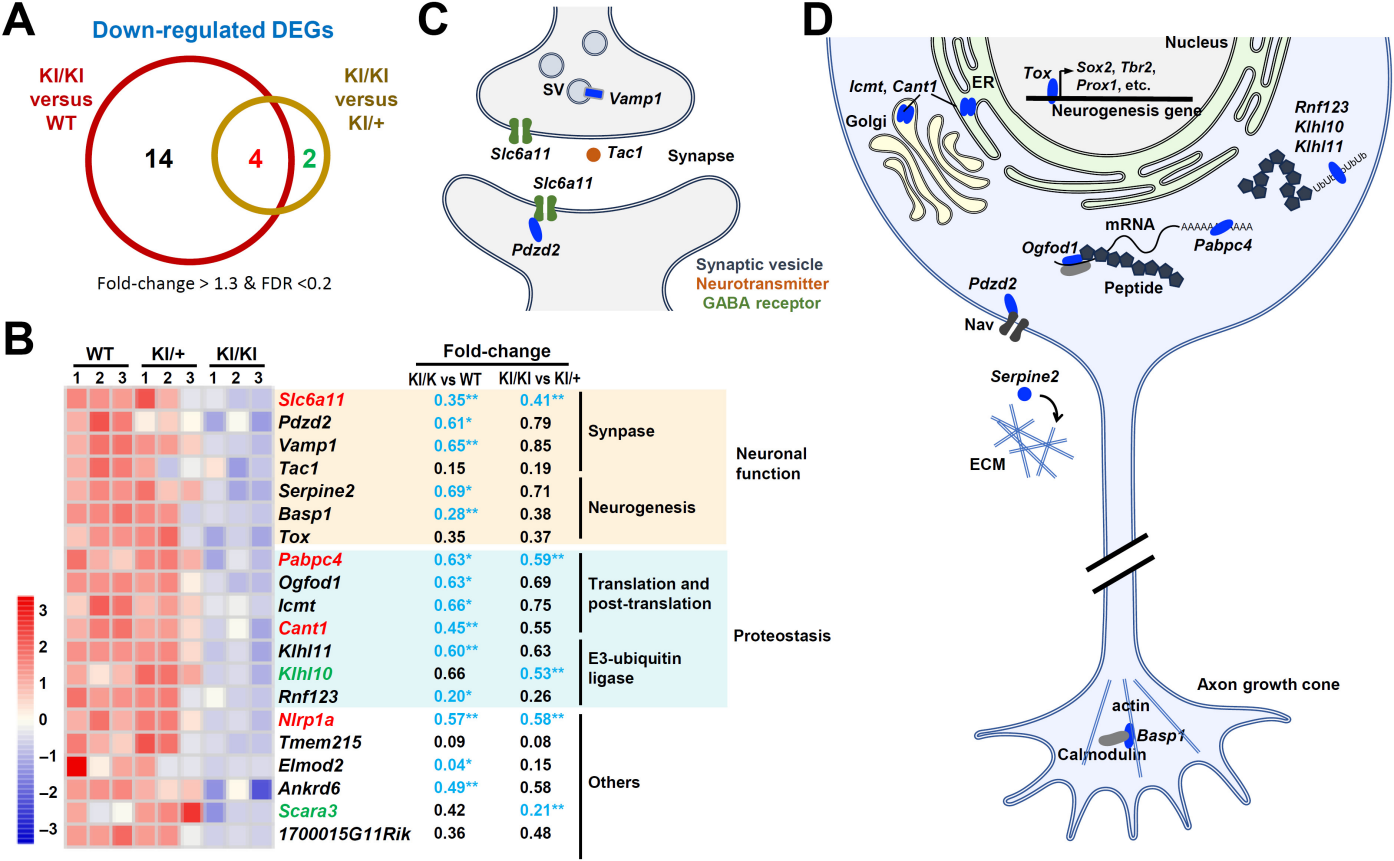
*Kcnd3* F227del down‐regulates a panel of differentially expressed genes (DEGs) that are involved in synaptic function and neurogenesis, as well as in translation and protein degradation. (A) Number of down‐regulated DEGs in Purkinje cells when comparing KI/KI versus WT, or KI/KI versus KI/+. (B) Heatmap of the mRNA levels of the DEGs affected by the Kcnd3 F227del mutant protein and their biological functions. The colors of the symbols correspond to the DEGs found in different comparisons in A. *FDR < 0.05; **FDR < 0.01. (C) Schematic diagram of the DEGs (*Vamp1*, *Slc6a11*, and *Tac1*) involved in synaptic function. (D) Schematic diagram of the DEGs involved in neurogenesis (*Serpine2*, *Basp1*, and *Tox*) and proteostasss (*Pabpc4*, *Ogfod1*, *Icmt*, *Cant1*, *Klhl11*, *Klhl10*, and *Rnf123*) in Purkinje cells. Blue, DEG‐encoded proteins that are down‐regulated in the KI/KI mice. Female mice at 6 weeks of age were used in this study.

To gain a better understanding of how the *Kcnd3* F227del mutation causes its phenotypic defects, we employed microdissection laser capture to isolate cerebellar tissues enriched with Purkinje cells from mice at 1.5 months of age (supplementary material, Figure S7) and performed RNA‐sequencing analysis. We identified 20 differentially expressed genes [DEGs; defined as fold‐change > 1.3 and false discovery rate (FDR) < 0.2] that were down‐regulated in the KI/KI mice compared with WT mice. Furthermore, there were six DEGs down‐regulated when KI/KI mice were compared with KI/+ mice (Figure 5A,B). However, no DEG was found when KI/+ mice were compared with WT mice. Based on the UniProt database, there were 15 down‐regulated DGEs that are mainly involved in the functioning of the synapse (*Slc6a11*, *Pzdz2*, *Vamp1*, and *Tac1*), the process of neurogenesis (*Serpine2*, *Basp1*, and *Tox*), translation and post‐translational regulation (*Pabpc4*, *Ogfod1*, *Icmt*, and *Cant1*), and the process of E3 ubiquitin ligation (*Rnf123*, *Klhl10*, and *Klhl11*) (Figure 5C,D). Interestingly, most of these biological processes are tightly linked to Purkinje cell function, specifically synaptic vesicle secretion and neurotransmission, as well as dysregulation of ER and Golgi functioning. Down‐regulation of E3 ubiquitin ligation is probably associated with the accumulation of Kcnd3 F227del mutant protein in the ER. In summary, our transcriptomic analysis at an early stage (1.5 months of age) of pathogenesis, prior to the degeneration of Purkinje cells at the age of 3 months (Figure 2A) and the development of an ataxia phenotype at the age of 4 months (Figure 1), provides evidence to gain insights into the molecular mechanism underlying the *Kcnd3* F227del mutation and how it causes SCA22.

### A loss‐of‐function *Kcnd3* null mutation did not cause a spinocerebellar ataxia phenotype

SCA22 is an autosomal dominant disease. Two possibilities are able to explain the phenotype that occurs in patients and in heterozygous KI/+ mice: (1) *Kcnd3* F227del is a gain‐of‐function mutation that exerts a dominant‐negative effect to interfere with the normal functioning of the WT protein; and (2) the ataxia phenotype is associated with haploinsufficiency, in which the 50% reduction in the normal protein is insufficient for complete cellular functioning. To differentiate the two possibilities, we generated a loss‐of‐function allele by deleting exon 2 from the *Kcnd3* gene, which contains the ATG initiation codon (supplementary material, Figure S8A). This genetic manipulation completely eliminated the expression of Kcnd3 protein (supplementary material, Figure S8B). At 4 months of age, both the heterozygous and the homozygous *Kcnd3* KO mice showed no overt phenotype in terms of growth and reproduction, and neither did they show any observable abnormalities including the ataxia phenotype (supplementary material, Video S3). This is in contrast to the obvious ataxia phenotype, namely motor coordination and balance defects that have developed at this age in the *Kcnd3* F227del mice (Figure 1 and supplementary material, Video S1). Furthermore, the *Kcnd3* KO (−/−) mice have no overt ataxia phenotype up to 9 months old. This provides convincing genetic evidence to demonstrate that the ataxia phenotype manifesting in human SCA22 patients and in heterozygous KI/+ mice is not caused by haploinsufficiency, but rather by *Kcnd3* F227del being a dominant‐negative mutation.

## Discussion

**Figure 6:**
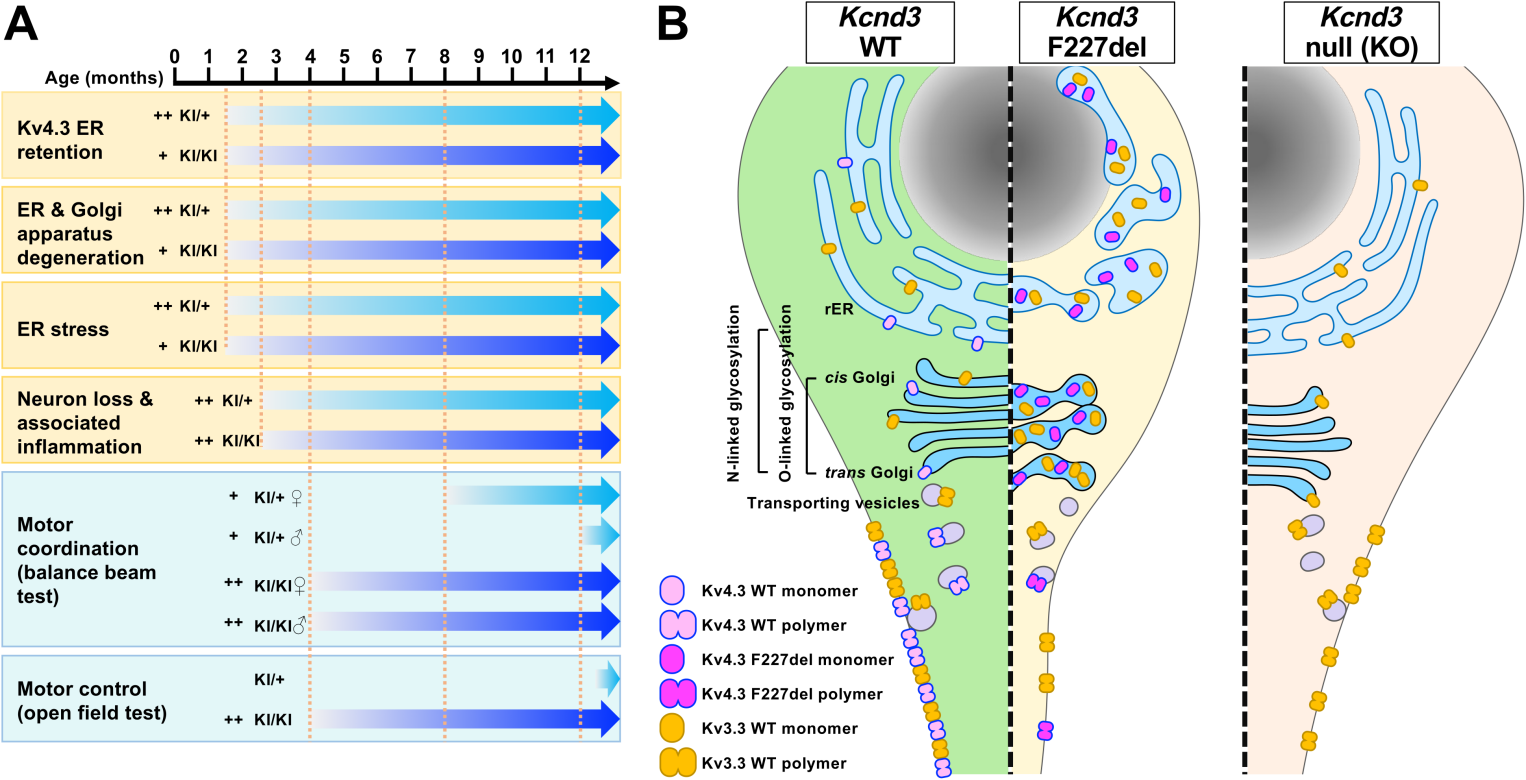
The Kcnd3 F277del mutant protein exerts a dominant negative effect via impairment of ER and Golgi apparatus functioning and jeopardizes membrane protein trafficking, thereby causing Purkinje cell dysfunction. (A) Summary of the cellular events and motor coordination phenotype as a function of age in the *Kncd3* F227del KI/+ and KI/KI mice. (B) Schematic illustration of the effects of *Kncd3* F227del mutation and *Kcnd3* null mutation in Purkinje cells.

In this study, we establish a KI mouse model carrying the *Kcnd3* F227del mutation to study the pathogenesis and molecular mechanism behind this mutation. Four significant findings are pinpointed. First, the *Kcnd3* F227del mutation causes an early onset of an ataxia phenotype that affects motor coordination and balance in both sexes. This mouse model exhibits a dosage‐dependent ataxia phenotype with a more severe defect being detected in homozygous KI/KI mice compared with heterozygous KI/+ mice (Figure 6A). Importantly, the KI/+ mice displayed the ataxia phenotype at around 8 months old, which mirrors the onset of ataxia in human SCA22 patients at 40–50 years old [8]. A loss of approximately 13% of Purkinje cells revealed by cresyl violet staining was found in KI/KI mice at 5 months of age, which is when an overt ataxia phenotype can be detected (Figures 1B,C and 2B). Regarding the degree of Purkinje cell loss in relation to the pathogenesis of an ataxia phenotype, previous studies have provided relevant information. In SCA6 mice, an approximately 50% loss of calbindin‐positive Purkinje cells was found when the mice developed ataxia [26]. In SCA2 mice, however, only about 10% loss of Purkinje cells was detected when the mice showed an ataxia phenotype [27]. Intriguingly, in ‘Moonwalker’ mice, the loss of Purkinje cells was found after the mice had developed severe ataxia [28]. Additionally, in SCA1 mice, the number of Purkinje cells was not decreased when the ataxia developed at 12 weeks old, but a 31% decrease of Purkinje cells was found at 24 weeks old [29]. Accordingly, the timeline between Purkinje cell loss and ataxia onset, as well as the percentage of Purkinje cell loss and the severity of ataxia, seems to be variable across different mutations. Thus, it is possible that the degeneration and a minor loss of Purkinje cells together with the concurrent presence of neuroinflammation (Figure 2F–I), as well as the electrophysiological changes present in the Purkinje cells, as suggested by Duarri *et al* [15], may all contribute to the ataxia phenotype in mice carrying the *Kcnd3* F227del mutation.

Second, the Kcnd3 F227del mutant protein is retained in the ER and Golgi, which in turn leads to activation of the UPR and to a severe trafficking defect affecting the movement of the mutant protein to its membrane destination. Intriguingly, pronounced damage to the Golgi within the Purkinje cells is the earliest ultrastructural manifestation of the mutation (Figure 6B). This is consistent with the conclusion from previous *in vitro* experiments showing that the *Kcnd3* F227del mutation causes ER retention of the mutant protein and this, in turn, results in significant suppression of the potassium current [8, 15]. Consequently, the above phenotype seems to compromise the membrane destination of other potassium channel proteins, such as Kv3.3.

Third, transcriptomic analysis revealed that at an early stage before the manifestation of the ataxia phenotype, many down‐regulated DEGs related to the *Kcnd3* F227del mutation can be identified. These are mainly involved in the functioning of the synapse and the process of neurogenesis, as well as translation and post‐translational regulation. All of these biological processes are tightly linked to the functioning and survival of Purkinje cells.

Finally, the ataxia phenotype is not present in heterozygous and homozygous *Kcnd3* KO mice carrying a loss‐of‐function null allele of *Kcnd3* up to 9 months of age. Consistent with this, a previously reported *Kcnd3* KO mouse model also had no overt neuropathological symptoms at young ages (up to 16 weeks old) [30]. Accordingly, *Kcnd3* F227del is a gain‐of‐function mutation that exerts a dominant‐negative effect in heterozygous mice. Furthermore, the mutant allele is detrimental to cerebellar functioning, which results in a dominantly inherited SCA disorder.

### Impaired trafficking of Kcnd3 F227del protein to its membrane destination and beyond

Accumulation of the Kcnd3 F227del mutant protein turns on ER stress and causes degeneration of the ER and Golgi, as well as impairing membrane protein trafficking. This working hypothesis is supported by the results presented here and by previous studies. For example, Valkova *et al* reported that Rer1 deficiency in Purkinje cells resulted in SCA phenotypes; this is caused by deficits in the membrane trafficking of Nav1.6 and Nav1.1 and by a reduction of spontaneous firing in Purkinje cells [31]. Another example is the study in Chinese hamster ovary (CHO) cells in which DPP10 (dipeptidyl aminopeptidase‐like protein 10) facilitates the membrane trafficking of Kv4.3 by protein–protein interaction and this is dependent on the glycosylation of DPP10. Mutations on the glycosylation sites of DPP10 disrupted the membrane trafficking of Kv4.3, which diminished current generation in CHO cells [32]. Thus, studying the role of DPP10 in Purkinje cells *in vivo* using the *Kcnd3* F227del KI mice will be very important in the future.

As an indirect effect, the transport of Kv3.3 to its membrane destination is significantly reduced in the cerebellums of *Kcnd3* F227del mice (Figure 4E–H). Genetic mutations in the *KCNC3* gene, which encodes Kv3.3, result in the pathogenesis associated with SCA13 [2]. Previous studies revealed that *Kcnc3* KO mice exhibit gait disturbance and have difficulty walking along a beam due to a prolonged action potential duration and the reduced firing frequency of Purkinje cells [17]. It is possible that a significant reduction in Kv4.3 and Kv3.3 at the plasma membrane may significantly reduce the firing frequency of Purkinje cells, thereby impairing the output inhibitory signaling, which then disrupts precise motor coordination.

### Perspective

We established the first preclinical mouse model for SCA22 by in‐frame deletion of the phenylalanine (F) at amino acid 227 of the *Kcnd3* gene. Previously, several preclinical SCA mouse models have been successfully applied to advance the progress of translational medicine. For instance, pharmaceutical corrections of ion channel functionality have been demonstrated to have beneficial effects in the preclinical models of SCA1, SCA2, SCA3, and SCA6, in which the disease‐driving proteins are linked with an expanded glutamine‐encoding CAG repeat [2]. Impressively, using a SCA1 mouse model, Lee *et al* discovered a novel mechanism upstream of the mutant ATXN1 and demonstrated that SCA1 is governed by region‐specific regulators of ATXN1; this has had a significant impact on the development of therapeutics [33]. Thus, the *Kcnd3* F227del mouse model has promise as another preclinical model for the development of innovative therapeutics that allow the treatment of human SCA22 via pharmaceutical approaches.

## Author contributions statement

B‐WS and T‐FT initiated the project, developed the working hypotheses and provided the conceptual framework. C‐HK, H‐CH, Y‐CT and T‐FT designed the experiments and interpreted the results. H‐CH and C‐HK carried out the mouse behavior experiments. C‐HK performed immunostaining, subcellular fractionation and cytokine array experiments. Y‐CT performed the transcriptomic analyses. C‐HK performed and analyzed the TEM results. Y‐CT and H‐CH prepared the figures and drafted the initial manuscript. T‐FT supervised all the experiments and wrote the final manuscript.

## Supporting information


Supplementary materials and methods

**Figure S1.** The generation of *Kcnd3* F227del knock‐in (KI) mice that display phenotypic defects affecting balance and coordination and resemble the changes in mobility found to affect SCA22 patients
**Figure S2.** Cytokine array analysis of cerebellar lysates from WT, *Kcnd3* F227del KI/+, and KI/KI mice
**Figure S3.** The *Kcnd3* F227del mutation causes obvious degeneration of the Golgi apparatus, mitochondria, and ER in the Purkinje cells at 6 weeks of age
**Figure S4.** The *Kcnd3* F227del mutation leads to the degeneration of the Golgi apparatus, mitochondria, and ER in the Purkinje cells at 4 and 6 weeks of age
**Figure S5.** Subcellular fractionation workflow and western blot analyses for autophagy markers in the cerebellums of WT, *Kcnd3* F227del KI/+, and KI/KI mice
**Figure S6.** The Kcnd3 F227del mutant protein was retained in the ER of neurons from the molecular layer of the cerebellum
**Figure S7.** Histological analysis of post‐laser captured cerebellar tissue
**Figure S8.** The *Kcnd3* knockout (KO) mice
**Table S1.** List of the antibodies used (referred to in Supplementary materials and methods)

**Video S1.** The gait of female WT, KI/+, and KI/KI mice at 4 months of age

**Video S2.** KI/+ and KI/KI mice displayed abnormal jumping behavior at a much higher frequency than did WT mice

**Video S3.** At 4 months of age, the heterozygous and homozygous *Kcnd3* KO mice show no observable ataxia phenotype

## Data Availability

The RNA sequencing data has been deposited in NCBI's GEO and are accessible through GEO Series accession number GSE280998 (https://www.ncbi.nlm.nih.gov/geo/query/acc.cgi?acc=GSE280998).
