# Supplementary material for: A dominant negative Kcnd3 F227del mutation in mice causes spinocerebellar ataxia type 22 (SCA22) by impairing ER and Golgi functioning
Source: J Pathol. 2024 Nov 19;265(1):57–68. doi: 10.1002/path.6368 (PMC11638663; doi:10.1002/path.6368)
Supplement: Supplementary file 1 — Supplementary materials and methods [file PATH-265-57-s001.docx]

**A dominant negative *Kcnd3* F227del mutation in mice causes spinocerebellar ataxia type 22 (SCA22) by impairing ER and Golgi function**

H-C Hung, J-H Lin, Y-C Teng *et al. J Pathol* <https://doi.org/10.1002/path.6368>

**Supplementary materials and methods**

Reference numbers refer to the main text list

*Balance beam test*

Our experimental setup for the balance beam test was modified according to Brooks and Dunnett [34] (Figure 1A). We recorded the walking behavior of mice as they traversed a 40-cm-long and 1-cm-wide wooden stick from one end to the other during daytime. Each mouse underwent three trials. We evaluated each mouse’s performance based on its ability to maintain steady and smooth walking across the beam. The following scoring criteria were used: mice with a perfectly steady and smooth walking manner received a score of 5; mice that slipped with one hind limb received a score of 4; mice that slipped with both hind limbs or slipped more than three times were scored 3; mice that belly-crawled across the beam were scored 2; mice that barely belly-crawled over half of the beam (20 cm) before falling were scored 1; and mice that were unable to balance on the beam or fell before completing half of the beam were scored 0. The scores were assessed by blinded examiners, and the average score for each mouse was subjected to statistical analysis.

*Open field test*

To conduct the open field test [34], a single mouse was placed inside a white box measuring 40 × 40 × 40 cm^3^ in a dark room illuminated only by dim red light. The mice were allowed to move freely within the box, and their behavior was recorded from an overhead perspective for a duration of 10 min (supplementary material, Figure S1E). The track of each mouse and the total distance covered by each mouse were analyzed using an EthoVision XT 13 (Noldus, Wageningen, The Netherlands). Additionally, the number of times each mouse engaged in rearing behavior was manually counted.

*Footprint analysis*

To mark mouse paw prints, the paws were dipped in nontoxic paint, the fore paws in blue and the hind paws in red. The mice were then placed on a sheet of drawing paper and allowed to walk freely through a 5-cm-wide and 100-cm-long tunnel, leaving their paw prints on the paper, three times.

The stride length was measured as the distance between the tops of the same palm between two steps using ImageJ (NIH, Bethesda, MD, USA). The results were obtained from WT, *Kcnd3* F227del KI/+, and KI/KI mice at 12 months of age. For each mouse, two fore paws and two hind paws were analyzed.

*Immunofluorescence staining*

The deeply anesthetized mice were perfused with PBS at room temperature, followed by ice-cold 4% PFA/PBS for tissue fixation. Whole brains were collected and immersed in 4% PFA overnight. The brains were then transferred into 15% sucrose/PBS for 6–12 h, which was followed by storage in 30% sucrose/PBS. Prior to sectioning, the brain tissues were soaked in OCT for 30 min, embedded, and snap-frozen using dry ice. The specimens were cryo-sectioned at –20 °C into 40 μm sagittal sections, which were then collected into six-well plates, and washed with PBS. The specimens were permeabilized with 0.3% Triton X-100/PBS for 15 min, followed by three 10-min PBS washes. Subsequently, the specimens were immersed in blocking solution (5% BSA/0.1% Tween 20/PBS) at room temperature for 30 min. This was followed by incubation with the primary antibody in blocking solution at room temperature overnight. After three 10-min PBS washes, the specimens were incubated with the secondary antibody in blocking solution at room temperature for 2 h. After another three 10-min PBS washes, the specimens were stained with DAPI (4',6-diamidino-2-phenylindole) at room temperature for 15 min and then washed twice with PBS for 10 min. The stained specimens were mounted with fluorescence mounting buffer (#S3023; DAKO, Glostrup, Denmark). The required images were captured using an Olympus FV10i (Olympus, Hamburg, Germany) or a Zeiss LSM700 (Zeiss, Oberkochen, Germany) confocal microscope. A list of the antibodies used is provided in supplementary material, Table S1.

*Cresyl violet staining*

After mouse perfusion as described, the whole brains were collected and immersed in 4% PFA overnight. Half of each mouse brain was embedded in paraffin and then sagittally sectioned at 5 μm. Paraffin sections were incubated at 65 °C for 1 h and then rehydrated. In brief, the specimens were immersed in cresyl violet working solution [Microscopy Cresyl Violet (acetate) for microscopy Certistain^®^, C5042; Sigma, St Louis, MO, USA] at room temperature for 40 min, and differentiated in 70% ethanol for 2–3 s, dehydrated, and mounted, following the manufacturer’s suggestions.

*Counting Purkinje cell number*

The images of immunofluorescence staining of calbindin were captured using an Olympus FV10i microscope. The number of calbindin-positive Purkinje cells in a whole mouse cerebellum was counted and normalized with the length of the molecular and granular layers’ junction underlying Purkinje cells, measured using ImageJ (NIH). The whole cerebellum section stained with cresyl violet was scanned using the MoticEasyScan digital slide scanning system (Motic Scientific, Schertz, TX, USA) with Motic EasyScanner software, and later images with high magnification, which covered the whole brain section, were captured using Motic DSAssistant software. For each image, the numbers of Purkinje cells showing normal and degenerated morphology were counted separately. Normalization to length was carried out as previously described for calbindin immunofluorescence staining.

*Western blotting*

The tissues were homogenized in lysis buffer and boiled for 5 min. Total lysates containing 8–15 μg of protein [as determined using the Bradford protein assay (Cat. # 5000006; Bio-Rad, Hercules, CA, USA)] were loaded onto 8–14% SDS-PAGE gels for electrophoresis. The separated proteins were subsequently transferred onto a PVDF membrane. After blocking with 5% non-fat milk/1% BSA at room temperature for 1 h, the membranes were incubated with the primary antibodies (supplementary material, Table S1) at 4 °C overnight. Following three washes with PBST, the membranes were incubated with the secondary antibodies at room temperature for 1 h. The immunoreactive bands were visualized using chemiluminescence (Cat. # 34580; Thermo Fisher Scientific, Waltham, MA, USA).

*Cytokine array*

The whole cerebellum was collected directly from deeply anesthetized 10-week-old mice. It was then homogenized in PBS with protease inhibitor cocktail (Cat. # 04693116001; Roche, Basel, Switzerland) at 4 °C. Next, the homogenates underwent two freeze/thaw cycles and were then centrifuged at 10,000 × *g* for 5 min to remove cell debris. The protein concentrations of the homogenates were measured using the Bradford protein assay (Cat. # 5000006, Bio-Rad). There were two samples for each genotype. In each sample, equal amounts of the cerebellum homogenate from a male and a female, namely 100 μg of homogenate from a male sample and 100 μg of homogenate from a female sample, were pooled to avoid a potential sex-specific effect on cytokine profiling. The cytokine array analysis (Cat. # ARY028; R&D Systems, Minneapolis, MN, USA) was carried out by following the manufacturer’s instructions. The immunoreactive dots were visualized by chemiluminescence (Cat. # 34580, Thermo Fisher Scientific). The signal intensities from the cytokine array were measured using ImageJ (NIH). The signal intensities of the positive controls on each array were used to normalize the signal intensities obtained from three arrays.

*Transmission electron microscopy* (*TEM*)

At 4 and 6 weeks of age, mice were deeply anesthetized and then perfused with ice-cold PBS before their whole brains were collected. The brain tissues were initially immersed in paraformaldehyde (Cat. # 15710; Electron Microscopy Sciences, Hatfield, PA, USA)/glutaraldehyde (Cat. # 16220, Electron Microscopy Sciences) buffer for primary fixation and subsequently sectioned into 1 mm × 1 mm × 1 mm cubes. Following this, the specimens underwent further fixation using osmium tetroxide buffer (Cat. # 19190, Electron Microscopy Sciences) and were then dehydrated in a series of ethanol concentrations, namely 70%, 85%, 95%, and finally 100%. The specimens were then sequentially immersed in propylene oxide (PO; Cat. # 20401, Electron Microscopy Sciences) at concentrations of 50%, 75%, and 100%. Subsequently, they were placed in a 1:1 mixture of PO and Spurr’s resin (Cat. # 14300, Electron Microscopy Sciences) before being transferred to 100% Spurr’s resin. Finally, the specimens were embedded in Spurr’s resin, and the ultrastructure of the Purkinje cells in each sample was visualized using a JEOL JEM 1230 electron microscope (JEOL, Kyoto, Japan).

*Laser microdissection of Purkinje cell-enriched tissue samples and subsequent RNA sequencing*

When 6 weeks old, the mice were deeply anesthetized and perfused with ice-cold PBS. Whole brains were collected and incubated in RNAlater™ (Cat. # AM7020; Invitrogen, Waltham, MA, USA) for 48 h. These brains were then snap-frozen in OCT and cryo-sectioned at –20 °C into 50 µm sagittal sections on PET membrane steel-frame slides (Cat. # 11505190; Leica, Wetzlar, Germany). The samples were stored at –80 °C until further processing [35]. To isolate cerebellar Purkinje cells and their adjacent tissue while avoiding RNA degradation, laser microdissection was performed using the Leica LMD7000 system within 2 h. Total RNA was extracted from the microdissected tissues using the RecoverAll™ Total Nucleic Acid Isolation Kit for FFPE (Cat. # AM1975, Invitrogen), following the provided protocol. RNA sequencing was conducted on triplicates of each genotype and gender, and this was performed by the Cancer Progression Research Center of National Yang Ming Chiao Tung University. Sequence alignment and read counting were carried out using Rsubread v2.8.2 [36]. Total counts per sample ranged from 4,032,357 to 1,915,339 (2,780,960 ± 525,392.8). Differentially expressed genes (DEGs) were identified using DESeq2 v1.34.0 [37] with the criteria for a DEG set at a fold-change greater than 1.3 and a false discovery rate (FDR) less than 0.2. Gene symbols were retrieved using ClusterProfiler v4.2.2 [38].

*Subcellular fractionation*

The protocol for subcellular fractionation was modified from a previous study [39]. In brief, 6-week-old mice were deeply anesthetized and perfused with ice-cold PBS. The whole cerebellum was collected and minced using scissors into 0.25 m SHE buffer (0.25 m sucrose, 1 mm EGTA, 3 mm HEPES, pH 7.5). The minced cerebellar tissues were homogenized using a 2-ml Dounce homogenizer (tight). The homogenates were then gravity-filtered through a 40-μm cell strainer (Cat. # 431750; Corning Inc., Corning, NY, USA) to remove unground tissues. The filtered homogenates underwent centrifugation at 1,000 × *g* for 10 min, which resulted in the collection of supernatant 1 and pellet 1. Supernatant 1 was further centrifugated at 10,000 × *g* for 10 min to collect supernatant 2, which was subsequently subjected to another centrifugation at 100,000 × *g* for 60 min to separate the cytosol and the microsomes (ER + Golgi). Pellet 1 was resuspended in 0.25 m SHE buffer and placed on top of 1.8 m SHE buffer to create a 0.25/1.8 m density gradient. After centrifuging at 71,000 × *g* for 90 min, the interphase (crude plasma membrane) and the pellet (nucleus) were collected. Each fraction was mixed with lysis buffer [50 mm Tris (pH 6.8), 100 mm NaCl, 1 mm EDTA, 0.2% Triton X-100, 0.5% sodium deoxycholate, 0.1% SDS, 1 mm NaF, 1 mm Na_3_VO_4_] to allow subsequent western blotting and analysis.

*Statistical analysis*

The results are presented as mean ± SD. Most experiments were analyzed using one-way ANOVA with Bonferroni’s correction for multi-comparisons test. The Kruskal–Wallis test was used to analyze the performance score of the balance beam assay and rearing times in the open field experiment. All statistical analyses were performed using Prism 8.0 (GraphPad Software, Boston, MA, USA).
