# Supplementary material for: A dominant negative Kcnd3 F227del mutation in mice causes spinocerebellar ataxia type 22 (SCA22) by impairing ER and Golgi functioning
Source: J Pathol. 2024 Nov 19;265(1):57–68. doi: 10.1002/path.6368 (PMC11638663; doi:10.1002/path.6368)
Supplement: Supplementary file 2 — Figure S1. The generation of Kcnd3 F227del knock‐in (KI) mice that display phenotypic defects affecting balance and coordination and resemble the changes in mobility found to affect SCA22 patients Figure S2. Cytokine array analysis of cerebellar lysates from WT, Kcnd3 F227del KI/+, and KI/KI mice Figure S3. The Kcnd3 F227del mutation causes obvious degeneration of the Golgi apparatus, mitochondria, and ER in the Purkinje cells at 6 weeks of age Figure S4. The Kcnd3 F227del mutation leads to the degeneration of the Golgi apparatus, mitochondria, and ER in the Purkinje cells at 4 and 6 weeks of age Figure S5. Subcellular fractionation workflow and western blot analyses for autophagy markers in the cerebellums of WT, Kcnd3 F227del KI/+, and KI/KI mice Figure S6. The Kcnd3 F227del mutant protein was retained in the ER of neurons from the molecular layer of the cerebellum Figure S7. Histological analysis of post‐laser captured cerebellar tissue Figure S8. The Kcnd3 knockout (KO) mice Table S1. List of the antibodies used (referred to in Supplementary materials and methods) [file PATH-265-57-s005.pdf]

A dominant negative *Kcnd3* F227del mutation in mice causes spinocerebellar ataxia type 22 (SCA22) by impairing ER and Golgi function

H-C Hung, J-H Lin, Y-C Teng *et al.* *J Pathol* https://doi.org/10.1002/path.6368

Supplementary Figures S1–S8

Supplementary Table S1

Figure S1

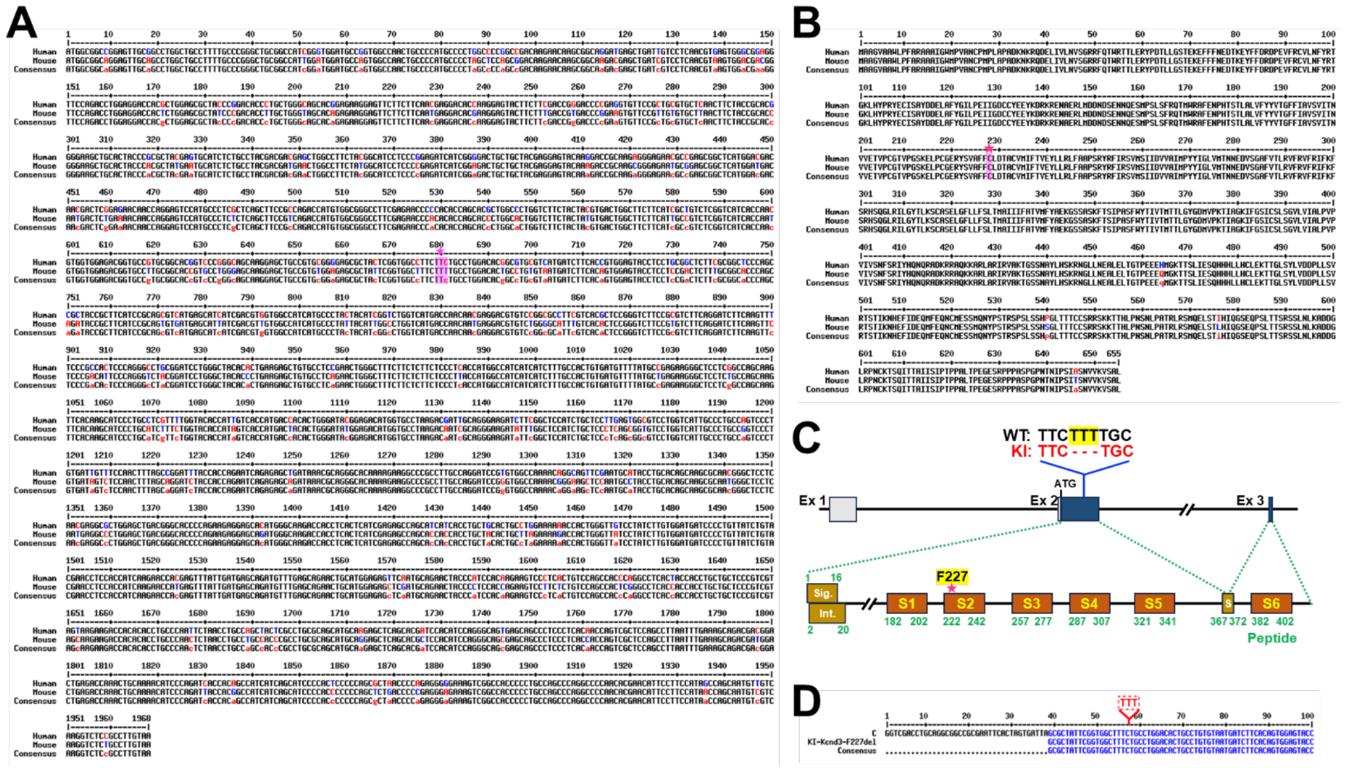

Figure S1. The generation of *Kcnd3* F227del knock-in (KI) mice that display phenotypic defects affecting balance and coordination and resemble the changes in mobility found to affect SCA22 patients. (A) A comparison of the coding sequences of human KCND3 and mouse Kcnd3 cDNA. The pink box and star indicate the F227 site. (B) A comparison of amino acid sequence of human and mouse Kcnd3 proteins. The pink box and star indicate the amino acid F227. (C) A schematic presentation of the *Kcnd3* F227del KI mouse. The upper part shows the *Kcnd3* gene at a genomic level, and the lower part illustrates the Kcnd3 protein domains. Ex, exon; Sig, signal peptide; Int, interaction with KCNIP2; S1–6, transmembrane region 1–6; S, selectivity filter. (D) Sanger sequencing confirmed the presence of the F227 deletion in the mouse *Kcnd3* gene.

Figure S1 (continued)

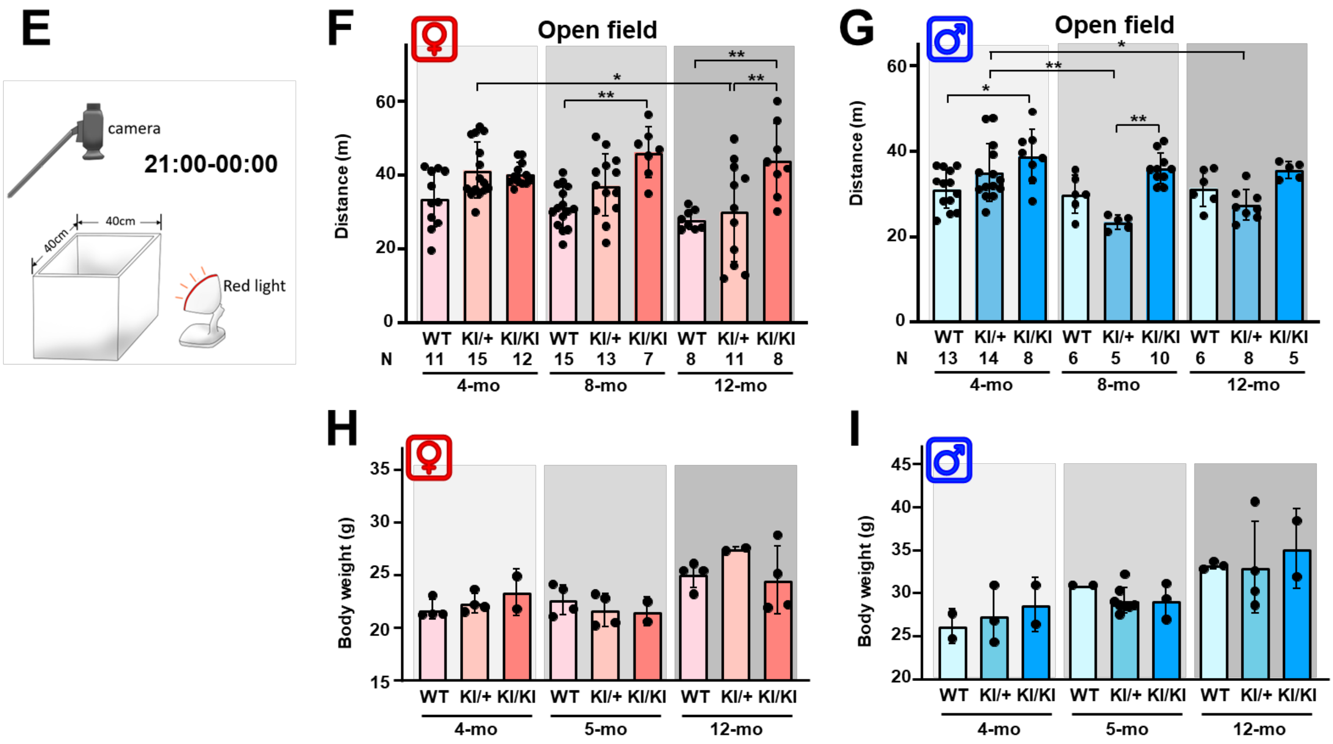

Figure S1 (continued). Generation of *Kcnd3* F227del knock-in (KI) mice, which display phenotypic defects affecting balance and mobility and resemble the changes in mobility found to affect SCA22 patients. (E) Device setup for the open field assay. Experiments were conducted during the night time (21:00 - 00:00) to stimulate the natural behavior of mice. (F,G) The total traveling distances of female and male WT, *Kcnd3* F227del KI/+, and KI/KI mice during the open field assay. (H,I) Growth curves for the female and male WT, *Kcnd3* F227del KI/+ and KI/KI mice at 4, 5 and 12 months of age. Mean ± SD. Statistical analyses were performed using one-way ANOVA corrected with Bonferroni's multiple comparison test to assess statistical differences. **p*<0.05; ***p*<0.01.

Figure S2

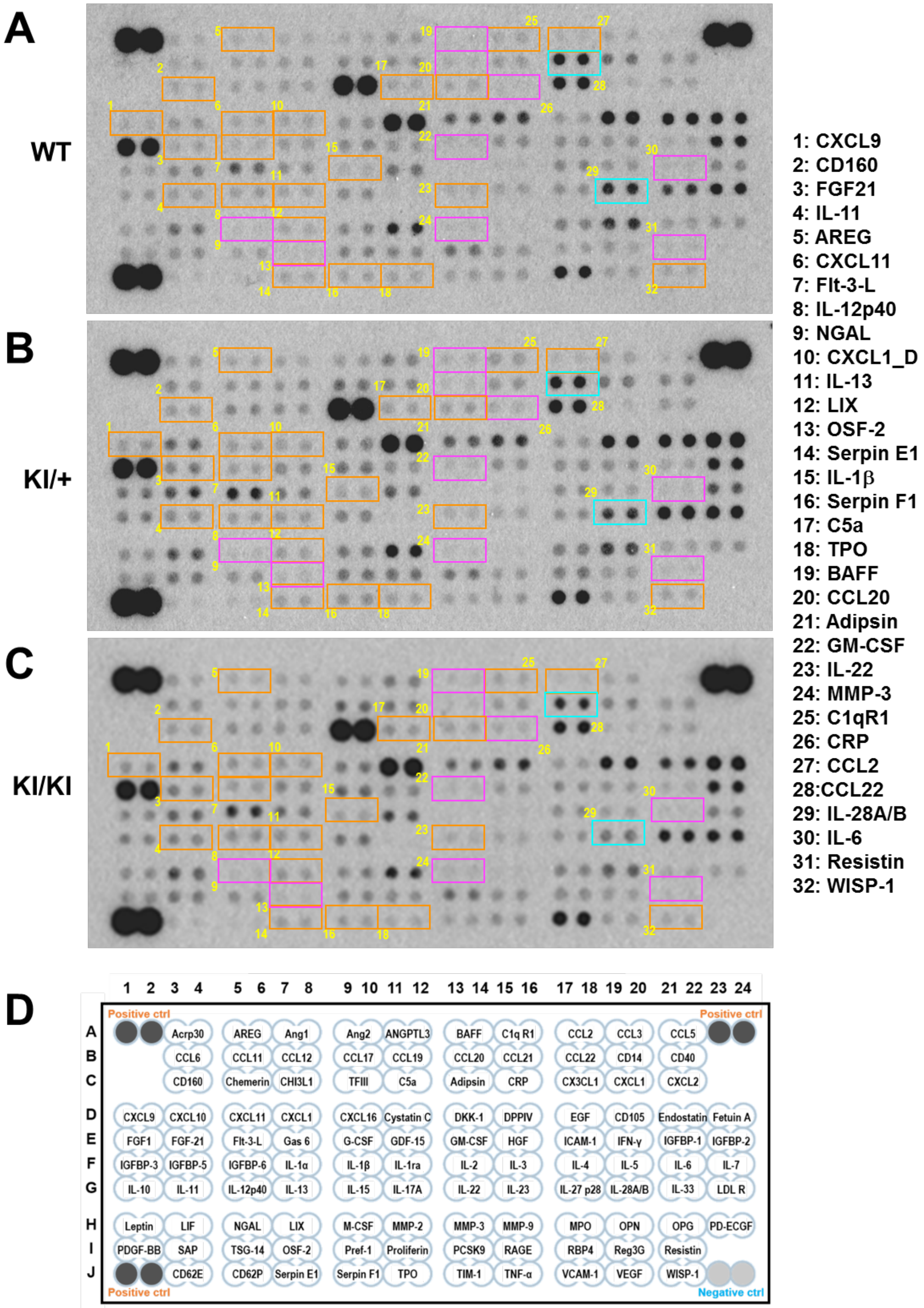

Figure S2. Cytokine array analysis of cerebellar lysates from WT, *Kcnd3* F227del KI/+ and KI/KI mice. (A–C) Images of the cytokine array assays using cerebellum lysates from (A) WT, (B) *Kcnd3* F227del KI/+ , and (C) KI/KI mice when 10 weeks old. (D) Map of the cytokines array.

Figure S3

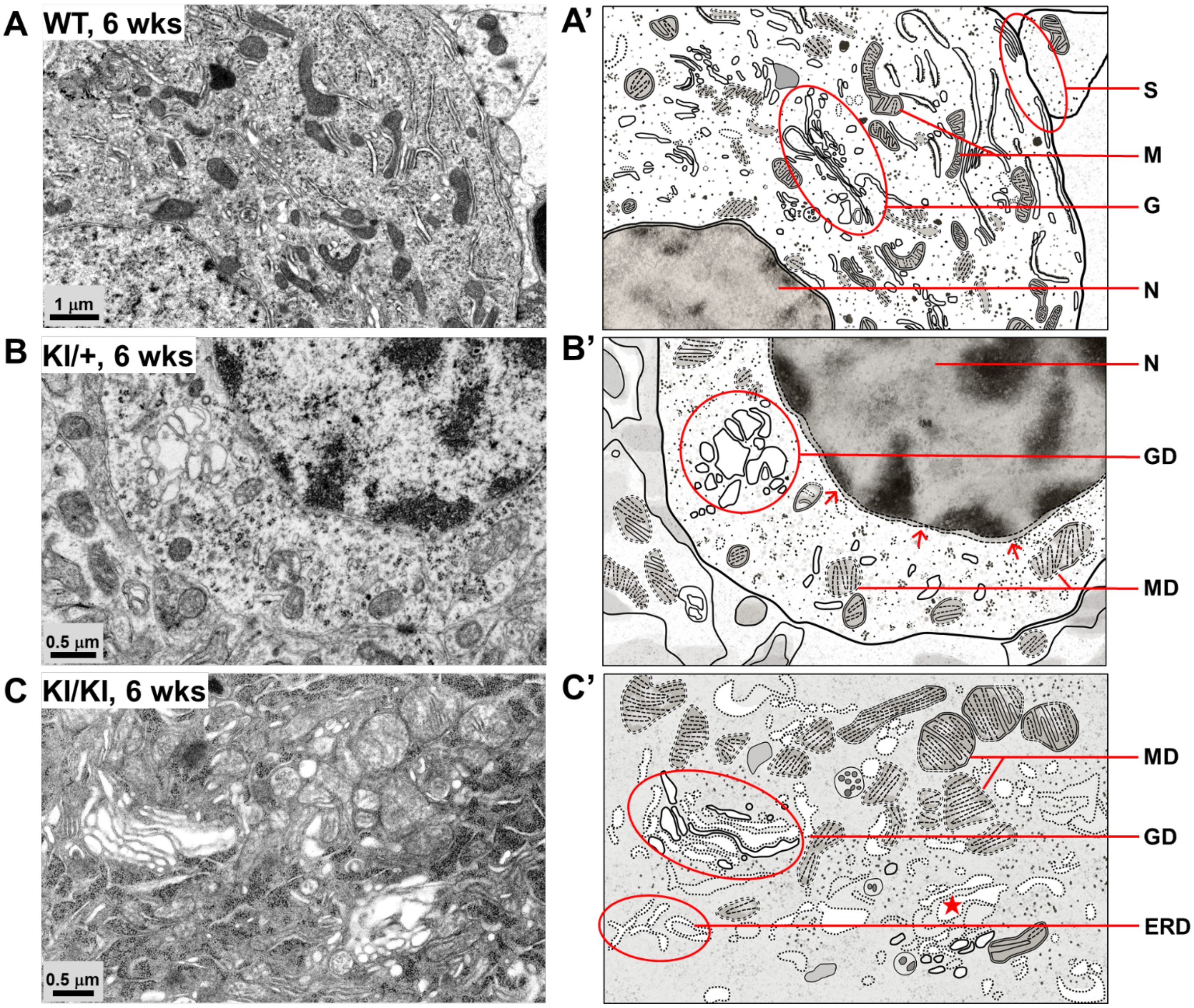

Figure S3. The Kcnd3 F227del mutation causes obvious degeneration of the Golgi apparatus, mitochondria and ER in the Purkinje cells at 6 weeks of age. (A)-(C) Representative TEM images of Purkinje cells in the WT (A), Kcnd3 F227del KI/+ (B) and KI/KI (C) mice. **(A')-(C')** Schematic representation of the TEM images (1:1 ratio) shown in A-C. Abbreviations: S, synapse; G, Golgi apparatus; GD, Golgi degeneration; M, mitochondria; MD, mitochondria dilation; N, nucleus; ERD, ER dilation; ★, ER, stress; red arrow, breakdown of nuclear envelope.

Figure S4

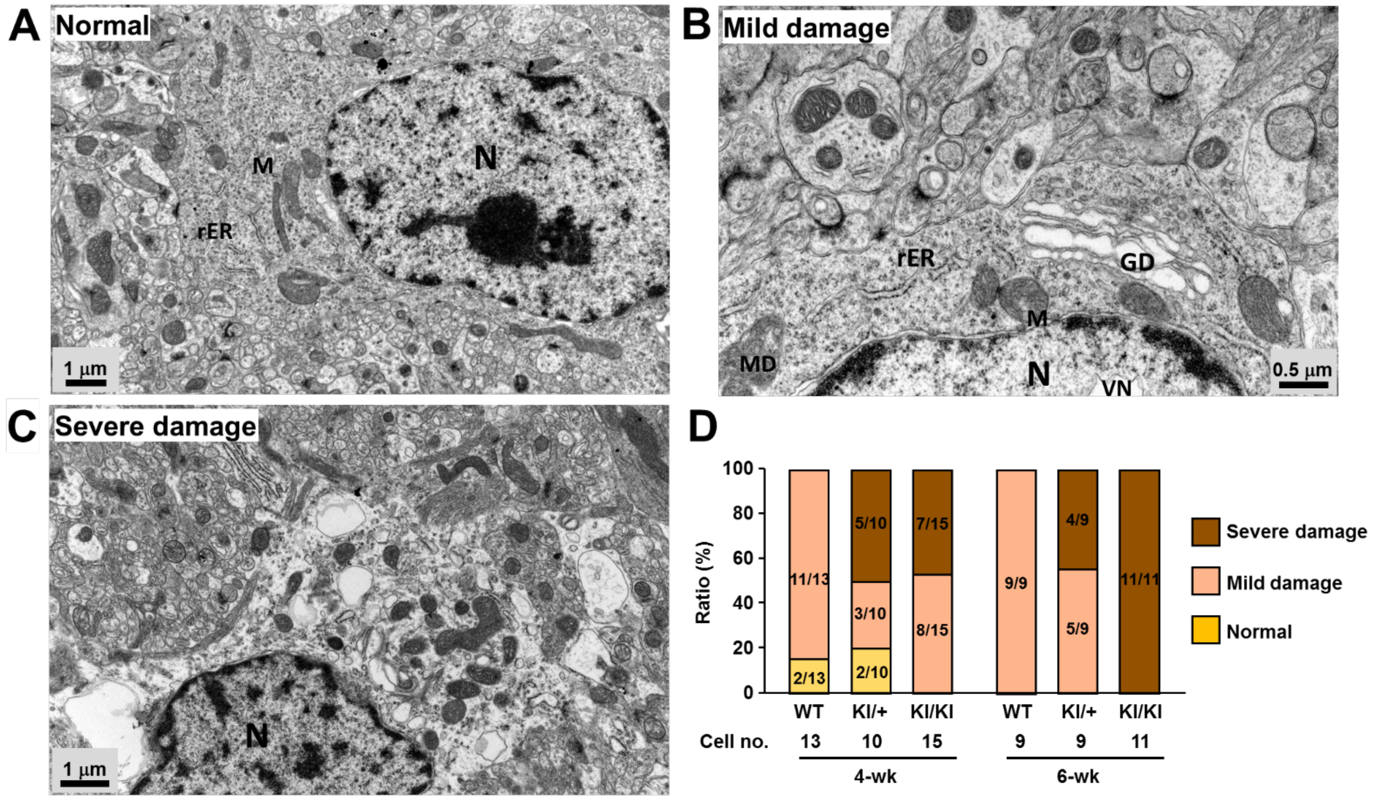

Figure S4. The *Kcnd3* F227del mutation leads to the degeneration of the Golgi apparatus, mitochondria and ER in the Purkinje cells at 4 and 6 weeks of age. (A) Representative image of Purkinje cell with normal morphology. (B) Representative image of Purkinje cell with mild damage, including Golgi degeneration (GD) and mitochondrial degeneration (MD). (C) Representative image of Purkinje cell with severe damage; this contains a large area of Golgi and mitochondrial degeneration, as well as necrotic cytoplasm. (D) The ratio of Purkinje cells exhibiting normal ultrastructure, mild damage, and severe damage in the cerebellum of the WT, *Kcnd3* F227del KI/+, and KI/KI mice when 4 and 6 weeks old. N, nucleus; rER, rough endoplasmic reticulum; VN, vacuole of nucleus.

Figure S5

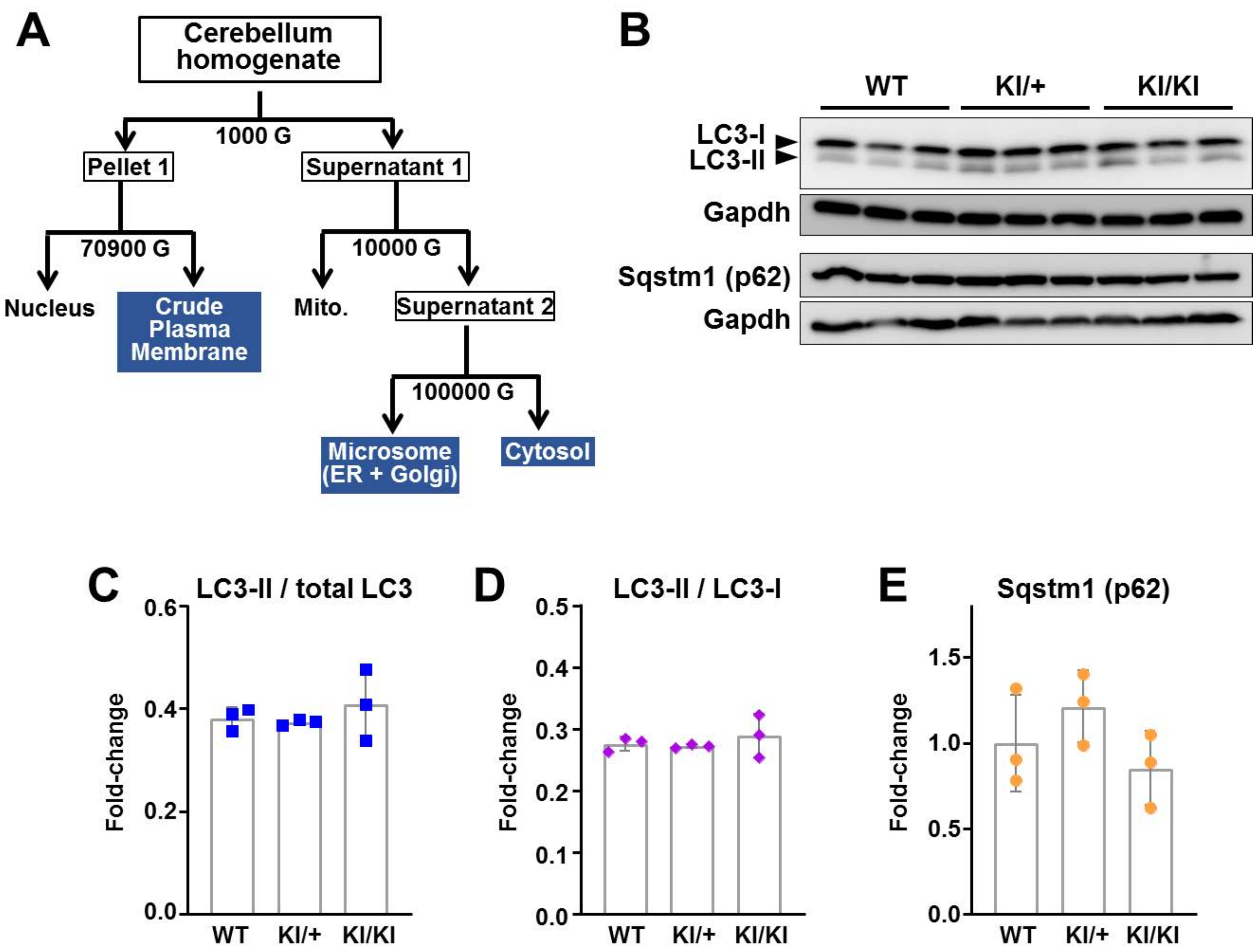

Figure S5. Subcellular fractionation workflow and western blot analyses for autophagy markers in the cerebellums of WT, *Kcnd3* F227del KI/+ and KI/KI mice.

(A) The protocol used for the subcellular fractionation experiment. (B) Western blot analyses for various autophagy markers, namely LC3-I and LC3-II, as well as p62, using tissue lysates obtained from WT, *Kcnd3* F227del KI/+ and KI/KI male mice at 6 weeks of age. (C) Ratio of LC3-II/total LC3 protein in panel B. (D) Ratio of LC3-II/LC3-I in panel B. (E) Protein level of p62 (normalized to Gapdh) in panel B.

Figure S6

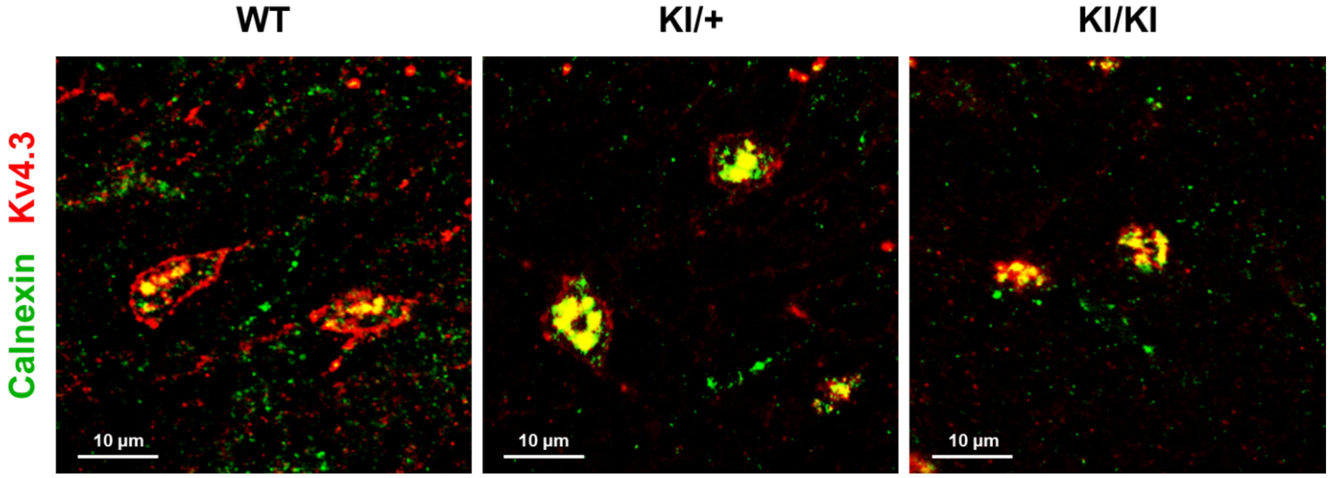

Figure S6. The Kcnd3 F227del mutant protein was retained in the ER of neurons from the molecular layer of the cerebellum. Immunofluorescence staining for Kv4.3 and Calnexin (marker for the ER) in the molecular layer of cerebellums of WT, *Kcnd3* F227del KI/+ and KI/KI female mice at 4.5 months of age.

Figure S7

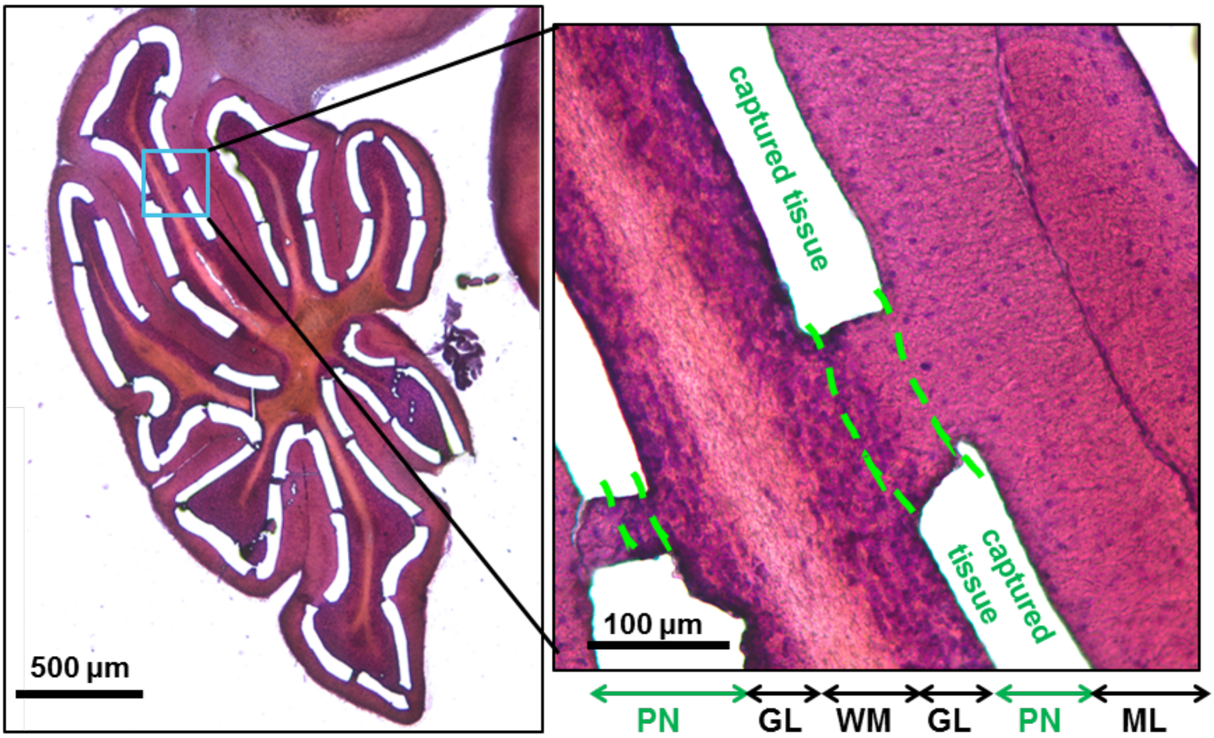

Figure S7. Histological analysis of post-laser captured cerebellar tissue. H&E staining revealed that the captured tissues mainly consist of Purkinje cell bodies. PN, Purkinje neurons; GL, granular layer; WM, white matter; ML, molecular layer.

Figure S8

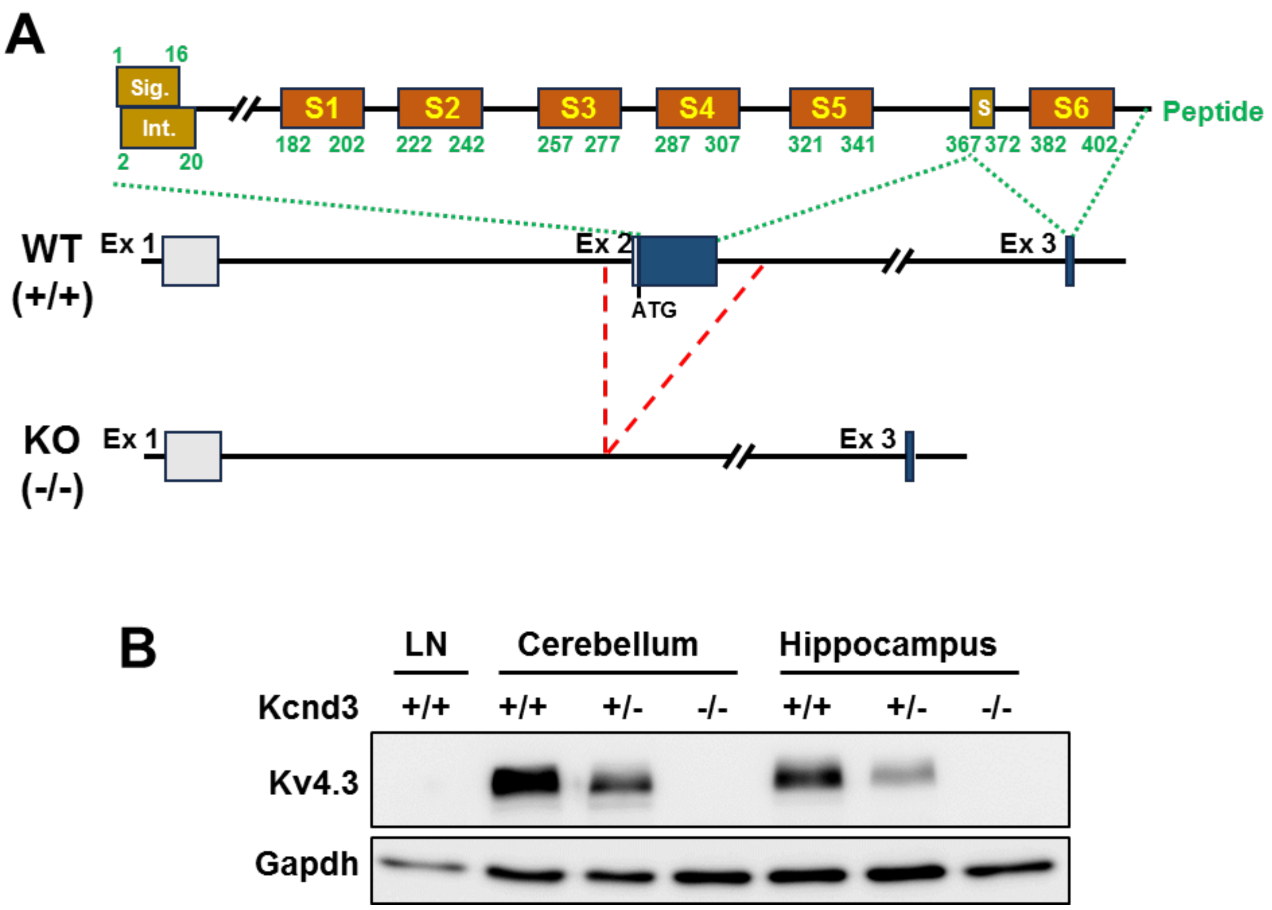

Figure S8. The *Kcnd3* knockout (KO) mice. (A) Genomic structure of the *Kcnd3* gene in the WT and *Kcnd3* KO mice. (B) Western blot analysis for the Kcnd3 protein using extracts obtained from the iliac lymph node (LN), the cerebellum, and the hippocampus of WT (+/+), heterozygous *Kcnd3* KO (+/-), and homozygous *Kcnd3* KO (-/-) male mice at 6 weeks of age. Ex, exon; Sig., signal peptide; Int., interaction with KCNIP2; S16, transmembrane region 1–6; S, selectivity filter.

Table S1. List of antibodies used.

Antibody	Supplier	Catalog number/clone number	Dilution	Experiment
Monoclonal anti-Calbindin-D-28K	Sigma (Saint Louis, MO, USA)	C9848/CB-955, monoclonal	1:1000	Immunofluorescent staining
Monoclonal anti-calnexin	Sigma	C7617/TO-5, monoclonal	1:1000	Immunofluorescent staining
Monoclonal anti-calnexin	Sigma	C7617/TO-5, monoclonal	1:5000	Western blotting
Anti-GAPDH Mouse mAb (6C5)	Sigma	MAB374/6C5, monoclonal	1:5000	Western blotting
Pan-cadherin whole antiserum	Sigma	C3678/Polyclonal	1:5000	Western blotting
Polyclonal anti-Kv4.3	Alomone lab (Jerusalem, Israel)	APC-017/Polyclonal	1:1000	Immunofluorescent staining
Polyclonal anti-Kv4.3	Alomone lab	APC-017/Polyclonal	1:3000	Western blotting
Polyclonal anti-Kv3.3	Alomone lab	APC-102/Polyclonal	1:5000	Western blotting
Iba1 Polyclonal Antibody	Invitrogen (Carlsbad, CA, USA)	PA5-27436/Polyclonal	1:2500	Immunofluorescent staining
Anti-phospho-eIF2α(pSer51) antibody	Sigma	SAB4504388/Polyclonal	1:500	Immunofluorescent staining
Anti-Mouse IgG H&L (Alexa Fluor® 488)	Abcam (Cambridge Biomedical Campus, Cambridge, UK)	ab150105/ Polyclonal	1:2500	Immunofluorescent staining
Anti-Rabbit IgG H&L (Alexa Fluor® 555)	Abcam	ab150074/ Polyclonal	1:2500	Immunofluorescent staining
Anti-Goat IgG H&L (Alexa Fluor® 647)	Abcam	ab150131/ Polyclonal	1:2500	Immunofluorescent staining
AMDEX™ Sheep Anti Mouse IgG-HRP	GE Healthcare (Chicago, IL, USA) Cytiva, Marlborough, MA, USA	GERPN4201	1:5000	Western blotting
Rabbit IgG HRP Linked Whole Ab	GE healthcare	GENA934	1:5000	Western blotting
